# Statistical analysis of software development models by six-pointed star framework

**DOI:** 10.1371/journal.pone.0264420

**Published:** 2022-04-01

**Authors:** Intakhab Alam, Nadeem Sarwar, Iram Noreen

**Affiliations:** Department of Computer Science, Bahria University, Lahore, Pakistan; GC Women University Sialkot, PAKISTAN

## Abstract

Software Development Process Model (SDPM) develops software according to the needs of the client within the defined budget and time. There are many software development models such as waterfall, Iterative, Rapid Application Development (RAD), Spiral, Agile, Z, and AZ model. Each development model follows a series of steps to develop a product. Each model has its strengths and weaknesses. In this study, we have investigated different software development process models using the six-pointed star framework. Six-point star is a framework of project management industry standards maintained by Project Management Body of Knowledge (PMBOK). A survey is designed to evaluate the performance of well-known software process models in the context of factors defined by the six-point star framework. The survey is conducted with experienced users of the software industry. The statistical analysis and comparison of results obtained by the survey are further used to examine the effectiveness of each model for the development of high-quality software concerning lightweight and heavyweight methodologies for small, medium and large scale projects. After exploring the results of all factors of the six-pointed star model, we conclude that lightweight methodology easily handles small-scale projects. The heavyweight methodology is suitable for medium and large-scale projects, whereas the AZ model, which is one of the latest models, works efficiently with both small-scale and large-scale categories of projects.

## Introduction

**Fig 1:**
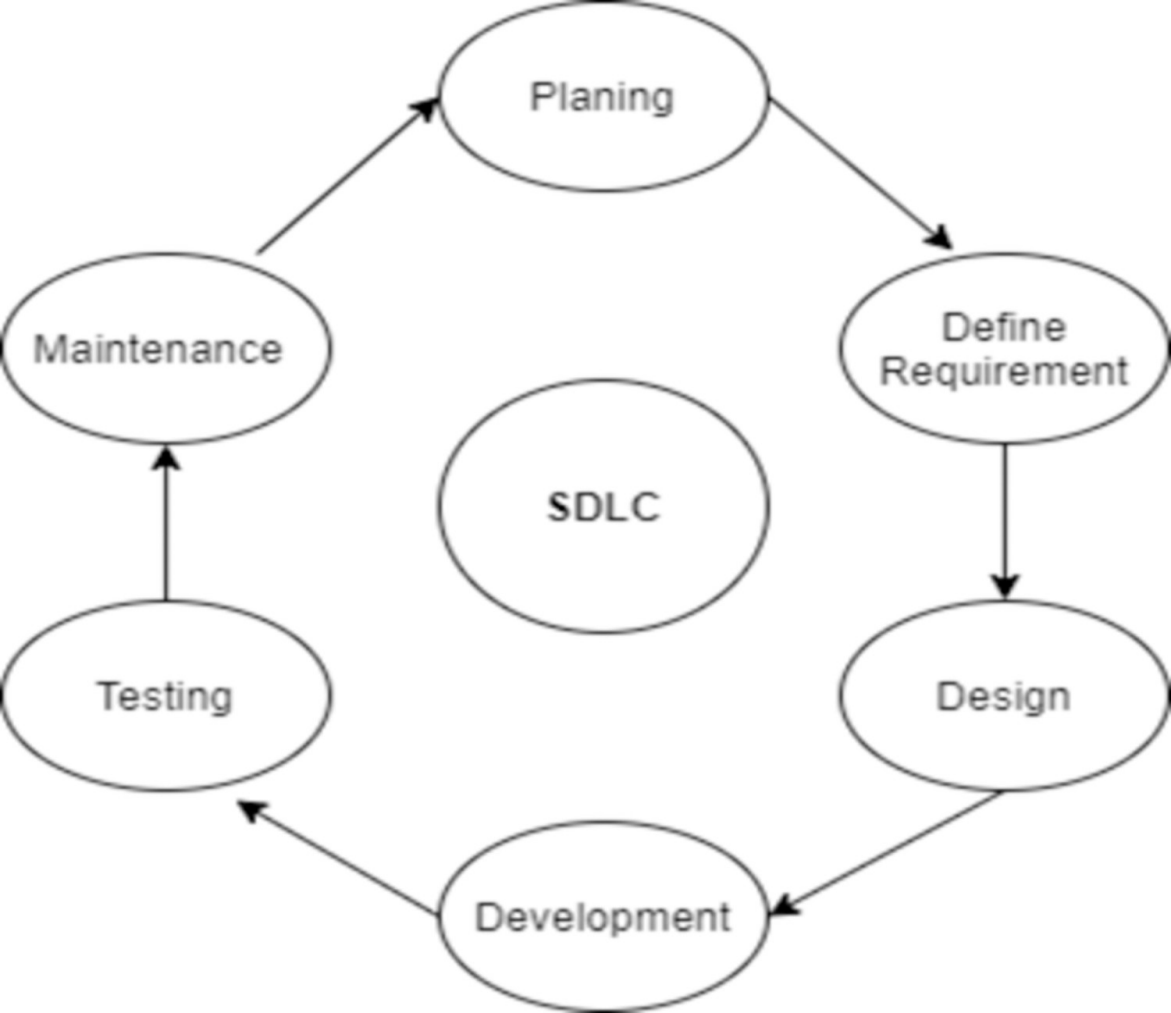
Software development life cycle.

Software development life cycle or SDLC is a process to develop software according to the step of series for designing, coding, testing, and finalizing the product [1]. There are many software development models according to the user as well as developer requirements [2]. The existing software development models, e.g., waterfall, iterative, agile, spiral, RAD, Z, and AZ model have their strengths and weaknesses. Some models provide the best results for short-term projects while others are beneficial for long-term projects. In some models, proper interaction between developer and client is highly emphasized such as agile whereas in other models, there is no proper developer and client interaction or it is limited to preliminary stages at managerial level. To achieve the desired output client interaction matters a lot in the development life cycle [3]. There are the following main phases of the software development life cycle as shown in Fig 1.

The first phase of the software development life cycle is the planning phase in which the senior team members interact with the client to collect the required information and wish list. Planning related to quality assurance and risk minimization is also identified in this phase. After completing the paling phase, the next phase is to define the requirements which are done with the help of software requirement specification (SRS). SRS document consists of all the product requirement which is necessary for the development of the product. Based on SRS, the developer proposes more than one design which are documented in document design specification (DDS). All the experts review this DDS and finalize a single design feasible for the product. The next phase is the development phase in which the developer uses specific high-level languages like C, C++, PHP, or Java to implement the desired features of the software product. The development phase is followed by the testing phase in which the complete developed product is tested to fix the errors and bugs. The last phase is the maintenance phase which consists of customer feedback. There are various software development models adopted by software teams according to their project needs [4]. These models are categorized as lightweight and heavyweight model based on multiple factors demonstrated in Table 1. Table 1 provides a comparison of both types of models [5].

**Table 1:** Comparison of heavyweight and lightweight methodologies [5].

Lightweight	Heavyweight
Less documentation	Heavy documentation (SRS)
Small team size	Large team size
People-oriented	Tool oriented
Nonpredictive approach	Predictive approach
Not critical	Extremely critical
Examples: Agile, AZ	Examples: Waterfall, Iterative, V Model

In this research, there is a comparison of both types of models concerning these models by applying PMBOK framework known as the six-pointed star model. Five models are included in this study which are categorized in lightweight and heavyweight methodologies in Table 1. In the literature review section, there is a detailed discussion of all these models.

In the past, the overall success of the development model has been determined by three factors (time, cost, scope) but in this research, we have adopted one of the methodologies of PMBOK known as the six-pointed star model. This methodology judges the success of development models on six factors (time, cost, scope, risk, resource, quality) which helps to improve the quality of the product.

The rest of the paper is organized as follows. In literature review section, there is a brief discussion of different software development models and recent trends for new software development models. In the research methodology section, there is a brief discussion of PMBOK six-pointed star framework. In the data collection section, there is a discussion of data collection and arrangement. In the result section, there is a brief discussion of results obtained by adopted methodology and in the last section, we conclude the study.

## Literature review

The main purpose of software engineering is to make reliable and high-quality software for users. Software development companies develop different types of software as per user requirements for the ease of users. For the development of software, the developer needs some model which is called the software development model. These models are applied according to international standards ISO/IEC 12270 [6]. The first model of SDLC was a fundamental part of software engineering because it provided a structure for different software development activities. Initially, speculation about the software development life cycles was that it’s a simple code but with the passage of time programming became complicated, consequently arising the need to upgrade the structure of software development [2].

The fundamental building blocks like the structured development method are available for the software development life cycle but the main challenge is how to improve the productivity and quality of the product. With the inclusion of supporting processes and iterative life cycle; the productivity and quality of product improved. IBM developed two of the main models which are called VIDOC and COMMAND. Based on the IBM ADP model manage the project quality and project management. After that, many models were developed by different companies [2].

Later, the importance of time-to-market of software to find out unclear and changing requirements has highly increased. In the case of any changes, the iterative model focuses mainly on the coding and testing phases. A new model was published called Boehm’s Spiral Model which includes requirement analysis in the iterations. After Boehm’s Spiral Model a new development model called rapid application development was published by martin [7]. The V model also called the Verification and Validation model was introduced with the simultaneous parallel testing feature during development phase. The major improvements in the software development life cycle are agile methodology and parallel plan-driven techniques. Nowadays, a great variety of software development models exist to develop the quality and reliable product. These methodologies are categorized as the lightweight methodology and the heavyweight methodology, each having their own pros and cons. The lightweight methodology is people-oriented while the heavyweight methodology is process-oriented [5].

A new software development model called the A-Z model is reported to be more efficient than all earlier models and according to its author, the A-Z model covers the drawbacks of all earlier models [8]. This model consists of three phases of communication, development, and product release. This is an intermediate methodology that works like a heavyweight as well as a lightweight method. The summary of related research work is described in Table 2.

**Table 2:** Literature review summary.

ID	Reference	Year	Main Idea	Limitations
1	Aslam et al. [9]	2019	Improve design pattern with PRIC technique.	PRIC technique does not focus on quality improvement of the software.
2	Azeem Akbar et al. [10]	2018	Improve requirement change management in GSD with Six-pointed star methodology.	The barrier of the RCM process is not discussed in GSD.
3	Mohit Kumar et al.[11]	2018	Importance of SDLC to develop high-quality software.	This research focuses on only two factors cost and quality and ignores another factor that is also necessary for the success of the project.
4	Azeem Akbar et al. [12]	2018	Different methodologies evaluate based on the six-pointed star model.	The six-pointed star model does not evaluate the development models based on GSD.
5	J. Yu [4]	2018	A detailed discussion of the characteristics of different software development models.	Not discuss the Software development models based on 4GT.
6	D. Galin [13]	2018	Software quality concept concerning different software development models.	Mainly focus of this research is related to quality and ignore other factors that’s a big limitation of this research.
7	Azeem Akbar et al. [8]	2017	A new technique is known as the AZ model for the development of high-quality software development with usability engineering.	A big limitation in the new AZ model is that it’s a limited agile model that’s why agile is a more preferable model to AZ.
8	H. S. Modi et al. [14]	2017	Comparative analysis of three different models with three different techniques.	The limitation of this research is that not all models discuss different techniques.
9	P. S. Helode et al. [15]	2017	Different software development techniques to develop software with its pros and cons.	The big limitation of this research is that there is no discussion of hybrid methodologies.
10	R. Kneuper [2]	2017	Sixty years history of software development life cycles and evaluation of new software development models.	Sixty years of development model explore but not using specific factors like (scope, budget, risk, resource, quality).
11	R. Arora et al. [16]	2016	Choose the right software development model according to user needs.	The limitation of this research is that not all models discuss because some models might be simulated using some tools.
12	M. A. Rather et al. [17]	2016	Different software process model discuses and explain that when the new model came into existence.	The limitation of this research is that different models explore without any specific perspective (quality, Cost) etc.
13	M. Mateen et al. [18]	2016	The AZ model fulfills the drawbacks of the previous model’s new methodology to develop software with quality.	A big limitation of the AZ model is that it’s not a completely customer-friendly model.
14	I. H. Sarker et al. [19]	2015	Survey of different development process models to choose the desired model according to requirement.	Not discuss the hybrid software development technique.
15	Alshamrani et al. [20]	2015	Comparative analysis of three software development models and discuss their pros and cons.	There is a big limitation that only three models compare and analyze their strengths and weaknesses.

Hence, most of the studies mentioned above are focused on two or three process models irrespective of the model’s category and comparison factors. Similarly other studies are mere survey papers which are not based on any survey or case study. Similarly, they are not evaluated using PMBOK framework of standards. Different software development models are used according to the requirements of clients to develop high-quality software [11]. Every development model consists of a series of steps to receive the desired output [17]. Different organizations follow different models for the development of projects. Some organizations use more than one model. The most widely used development models in the software industry are described as follows.

### Waterfall model

The first sequential process model is called the waterfall model which was developed by Royce in 1970. This model belongs to the heavyweight model category because there is a need for proper documentation. This model consists of steps of series to develop a well-defined product [21]. There are the following steps of the waterfall model communication, planning, designing, construction, and deployment. It is a very simple and easy-to-use model, but the main disadvantage of this model is that there is no back-tracking possible in this model. One phase is complete then the next phase starts that’s why this model is not preferable for long-term projects [14]. The waterfall is suitable for where requirements are clear, technology is defined, and no confusing requirements exist. This model is easy to manage. All stages of this model are clearly defined. However, this model is not feasible for complex, dynamic, and high-risk projects because it does not accommodate changing requirements [10].

### Iterative model

This model consists of four phases (requirement, analysis, design, coding) and the product is delivered in the form of iterations. This model also belongs to the heavyweight model category because this model tool oriented and there is a need for proper documentation. If something is missing it can be accommodated in the next iteration and so on [22]. The iterative model is suitable where the requirement evolves with time, domain new, and project lengthy. In this model, the product delivery rate is faster, and prioritize requirements develop earlier. But the total cost of the process is high [15,23].

### Agile model

In agile methodology, there is no need for proper planning but there is clarity of future work. The backbone of agile methodology is close customer interaction, a friendly environment of client and developer, and support for changing requirements [24]. The agile model relates to lightweight model category. The agile model is suitable where the least documentation is required, and the geographical location is also the same. In this model, the daily conversation of the developer and client improves the satisfaction of the customer and easily accommodates changes. Its product delivery rate is fast. Due to no documentation [13] the project details are not very clear for future enhancement and scalability. This model is suitable for small to medium-scale projects.

### V model

This model is like the waterfall model, also known as the verification and validation model. Each phase completes before the next phase starts and parallel testing is the main feature of the V model [19]. The V model is suitable for small and medium-scale projects also V model is preferred when expert-level technical team and resources are available. In this model, parallel testing ensures to clear all bugs and defects at the early stages. However, this model is not much flexible for changing requirements because this model relates to heavyweight model category. If any change is required, then the SRS document must be updated accordingly.

### AZ model

AZ-model is one the latest model that works on the lightweight and heavyweight methods [18] but it is mostly suitable for lightweight model category. This model consists of three phases (customer interaction, development, product delivery). In this model, there is the concept of usability testing and time-boxing which can enhance the quality of the developed product [8]. The AZ model is suitable for small as well as large-scale projects. This model is also suitable where limiting work is in progress. This model provides high-quality software. The main feature of this model is usability testing. This model is people as well as process-oriented. The focus of this model is client satisfaction but the client interaction in this model is limited. Table 3 summarizes the pros and cons of the aforementioned software development model.

**Table 3:** Pros and cons of software development life cycle.

Models	Pro	Cons
Waterfall	◾ Easy to manage. ◾ Simple and easy to use.	◾ Not flexible. ◾ Not suitable for complex projects.
Iterative	◾ Results were obtained earlier. ◾ Risk managed easier. ◾ Good for large-scale projects.	◾ Costly model. ◾ More resources are required.
Agile	◾ Easy to manage. ◾ Flexible model. ◾ No planning is required.	◾ Not suitable for complex projects. ◾ More risk due to lack of documentation.
V Model	◾ Simple and easy to use. Good for small-scale projects.	◾ No good for complex projects. ◾ High risk.
AZ	◾ Good for light and heavyweight methodologies. ◾ Usability testing feature improves the performance of developed software. ◾ The concept of timeboxing improves efficiency.	◾ Customer interaction is limited.

## Research methodology

**Fig 2:**
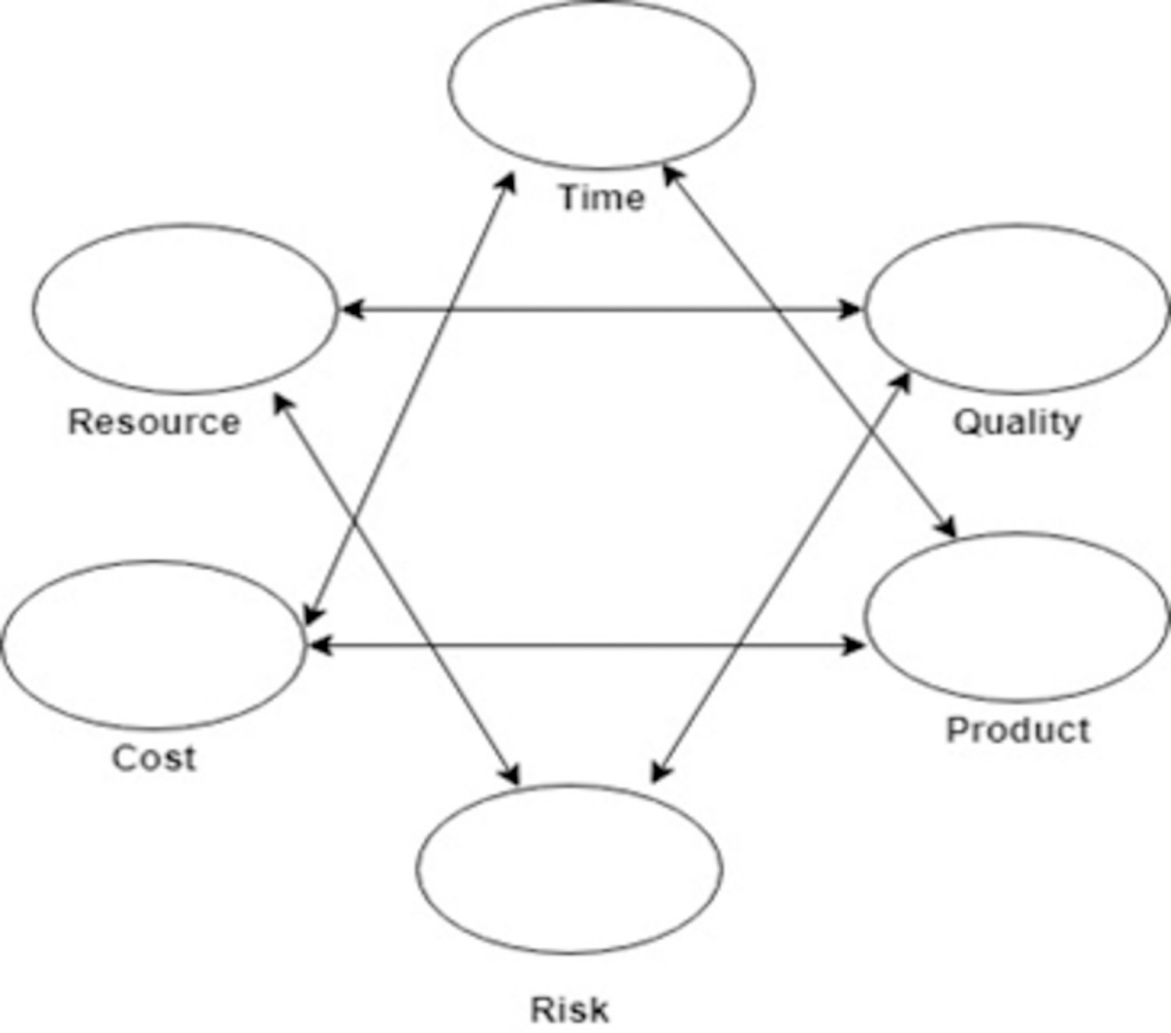
Six-pointed star framework of project management body of knowledge [25].

The project management body of knowledge (PMBOK) is a framework of standards, conventions, processes, best practices, terminologies, and guidelines that are accepted as project management industry standards. The PMBOK refers to the five process steps of project management: initiating, planning, executing, controlling, and closing [8]. One of the models of PMBOK is called the Six-pointed star framework. Traditionally the software success is evaluated using three factors (time, cost, scope). However, now overall success can be measured with the help of a model by the project management body of knowledge called the six-pointed star framework. This model consists of six factors (scope, budget, time, resource, risk, quality) in star formation as shown in Fig 2 [25].

**Fig 3:**
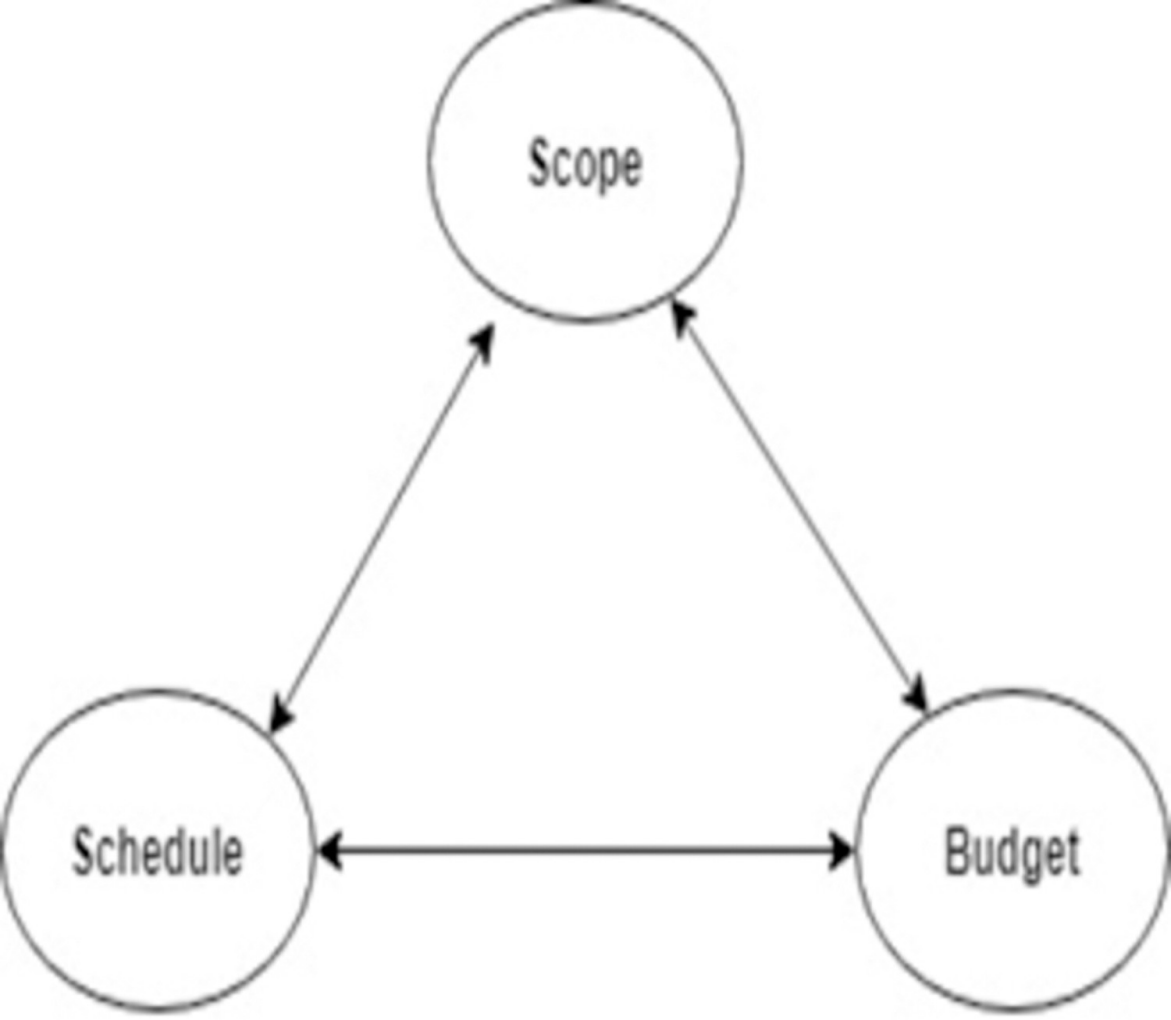
First triangle of PMBOK six-pointed star framework [25].

**Fig 4:**
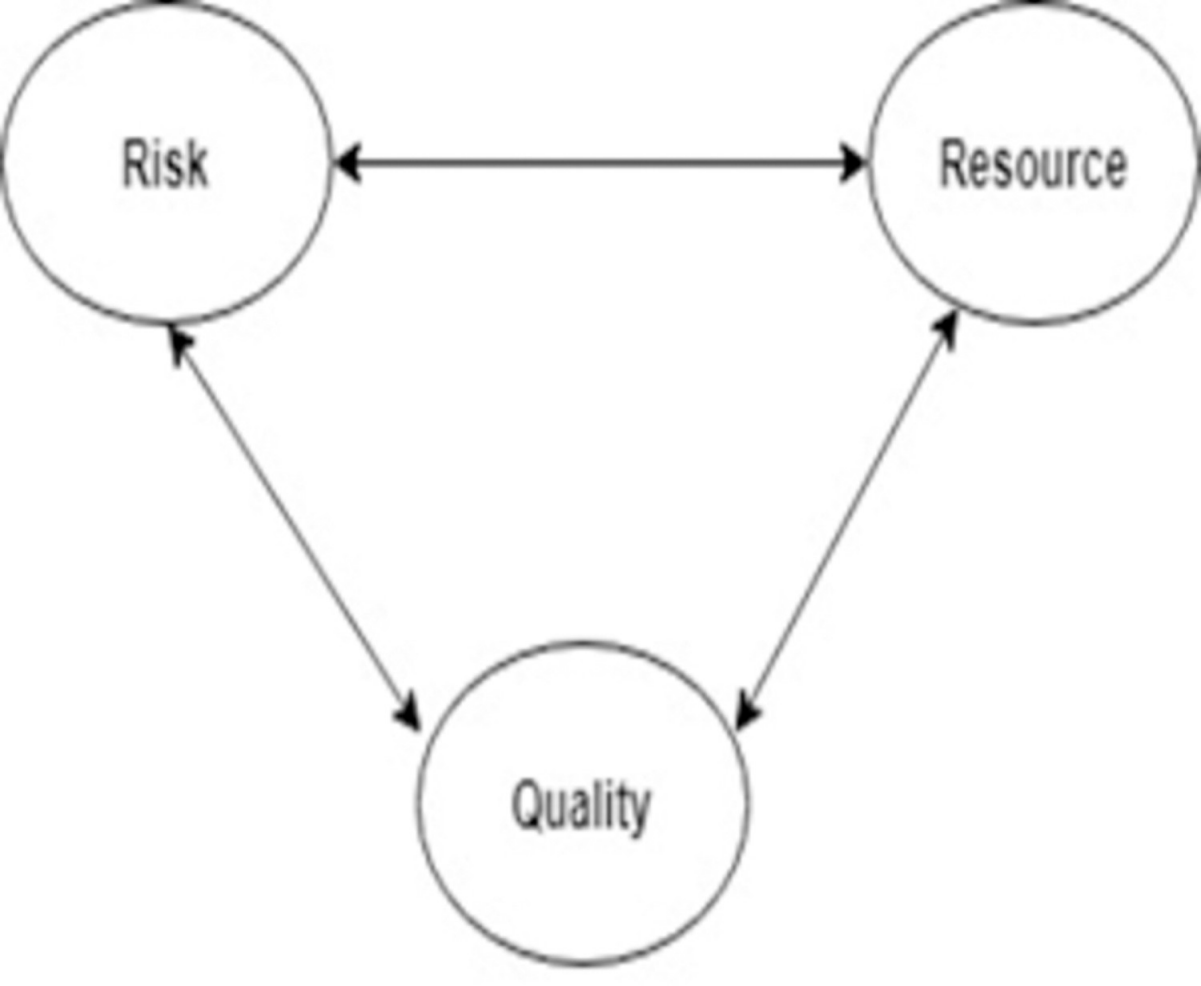
Second triangle of PMBOK six-pointed star framework [25].

This model is divided into two triangles. The first triangle consist of (scope, schedule, budget) which are used as input/ output factors and the second triangle consists of (risk, resource, quality) known as process factor [26] shown in Figs 3 and 4. We applied this method to different software development models and performed a comparative statistical analysis to investigate that which model is more efficient [22].

## Data collection

Data is collected with the help of a survey form. This survey form consists of three phases. The first phase collects the personal information of the respondent; the second phase comprehends the general information about the organization and the last phase encompasses questioner designed based on the factors of the six-pointed star method. Moreover, a clause of an ethics statement is also included about informed consent to make sure the respondent that their information will be kept confidential and will be used only for research purposes. This survey started from July 2020 to November 2020 and twenty-six organizations participated in this survey resulting in a collection of 31 responses. Some organizations fill in more than one survey form. Responses were collected based on the Likert scale (strongly agree, agree, neutral, disagree, strongly disagree) [25]. If any respondent follows more than one model, then they submit two survey forms, i.e., one for each model. The results were obtained in numeric format. We summarized these results and applied statistical graphical techniques by using well-known statistical tools on the collected data. The data relating to the survey (questionnaire forms, respondent response forms) is available at the Google drive link [27]. According to general information, our respondents belong to different software organizations having experience of 3–5 years with both lightweight and heavyweight types of models in both small and/or large organizations. Most of the respondents informed us that the two factors (budget and quality) are important when they are adopting a methodology for developing software. The respondents of organizations give their opinion (with the help of the Likert scale) about different development models. Respondents give their opinion about the selected model in lightweight as well as heavyweight methodology. The distribution of software development models is shown in Table 4 in a supporting file. In Table 4, there is a distribution of software development models which is selected by developers of different software houses. When respondents fill the survey form, they select a specific development model that is applicable in their organization. According to the survey mostly respondents prefer the waterfall model. The agile and iterative model is preferable to the waterfall model. In the last V and AZ model selected by respondents.

**Table 4:** Distribution of software development models.

Model	Selected
Waterfall	11
Iterative	10
V	7
Agile	10
AZ	7

### Ethical concerns

To prevent the association of any ethical concerns to this research project, certain measures were adopted, i.e., the research participants were asked about their consent before participating in this research study. Moreover, no personal details of the research participants were collected other than their email IDs, and the research participants were informed about this act. All in all, the researcher practiced a high level of morality and ethics to meet and support the confidence of the research participants. Moreover, a post-graduate research project evaluation and ethics committee consisting of three senior Ph.D. members have also approved the ethical review form of the study.

During the data collection, respondent shares their opinion about different software development methods [10]. Most respondent suggests a lightweight method is best for small and medium scale projects and a heavyweight method best for large-scale projects. The small medium and large scale projects are categorized depending on some important factors as shown in Table 5.

**Table 5:** Categorization of small medium and large scale projects.

Factors	Small Scale Projects	Medium Scale Projects	Large Scale Projects
Duration	Less than six months	Six to twelve months	More than twelve months
Budget	Less than 100,00 $	100,00$ - 500,00$	Greater than 500,00$
Team Members	Fewer than 5 people	5–20 people	Greater than 20 people
Integration	Minimal with other business units	Moderate with other business units	Significant with other business units
Impact	Fewer than 25 end-users	25–250 end-users	More than 250 end-users

The questioner form (implemented on both methodologies) is based on six factors of the project management body of knowledge is shown in Table 6. Table 7 shows the numeric value of the Likert scale score for the collection of respondents’ responses.

**Table 6:** Questioner form based on six-pointed star methodology.

Factors	Questions
Schedule	Questioner related to schedule: • Gratifying project requirements. • Task manged according to schedule. • Awareness of project status by the project team.
Scope	Questioner related to scope: • Overall decisive scope of the project. • Team members have clarity in scope.
Budget	Questioner related to budget: • Project accomplished in the decided budget. • Return Good or not.
Risk	Questioner related to risk: • Management of risk. • Meet business aspiration.
Resource	Questioner related to resource: • Availability of resources. • Utilization of resources.
Quality	Questioner related to risk: • Statisfaction of client. • Successfully Accomplish.

**Table 7:** Five points Likert scale for the collection of respondent response.

Response	Score
Strongly Disagree	0
Disagree	1
Neutral	2
Agree	3
Strongly Agree	4

## Results

The literature reveals that the six-pointed star methodology only applies to the AZ model that enables the enhancement of the quality of software [9]. In this paper, we apply the six-pointed star methodology to lightweight and heavyweight software development models and perform a statistical analysis and compare the results of different models to check the efficiency of the development process. The results based on the factors of selected methodology and these results are calculate with the help of Eq 1.


$$<Display\_Math>Score\;=\;\frac{\sum n}{n}$$ 1

Where *n* is a number of responses.

Next section provides the comparative graphical representation of both methodologies based on the six-pointed star model. Results comparisons of both methodologies are discussed in the following subsections.

### Lightweight vs lightweight

#### Agile vs AZ

**Fig 5:**
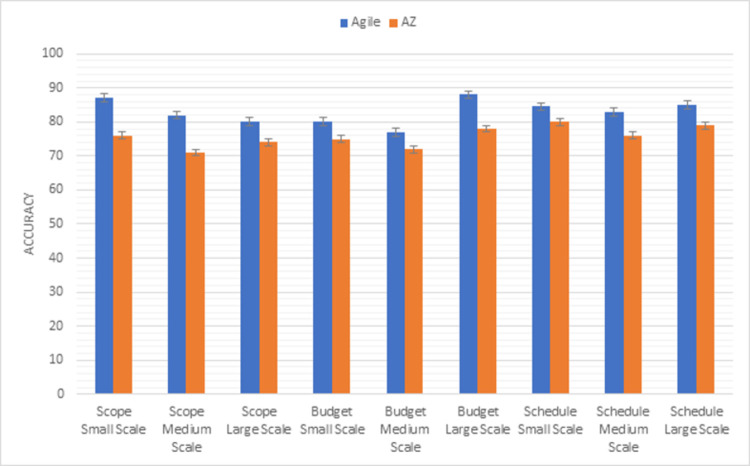
Graphical representation of agile vs AZ (first triangle) results.

Agile is also one of the best processes to use an adaptive approach where there is no need for a detailed documentation process. Customer interaction is one of the best features of an agile model. In the AZ model, customer interaction is limited only in the first phase which is the weak point of this model as compared to the Agile model. Comparison results of the agile model with the AZ model using the first triangle of PMBOK methodology factors (scope, budget, schedule) are shown in Fig 5. The agile model is more preferable in the scope of budget and schedule factors in (small, medium, and large) scales projects.

**Fig 6:**
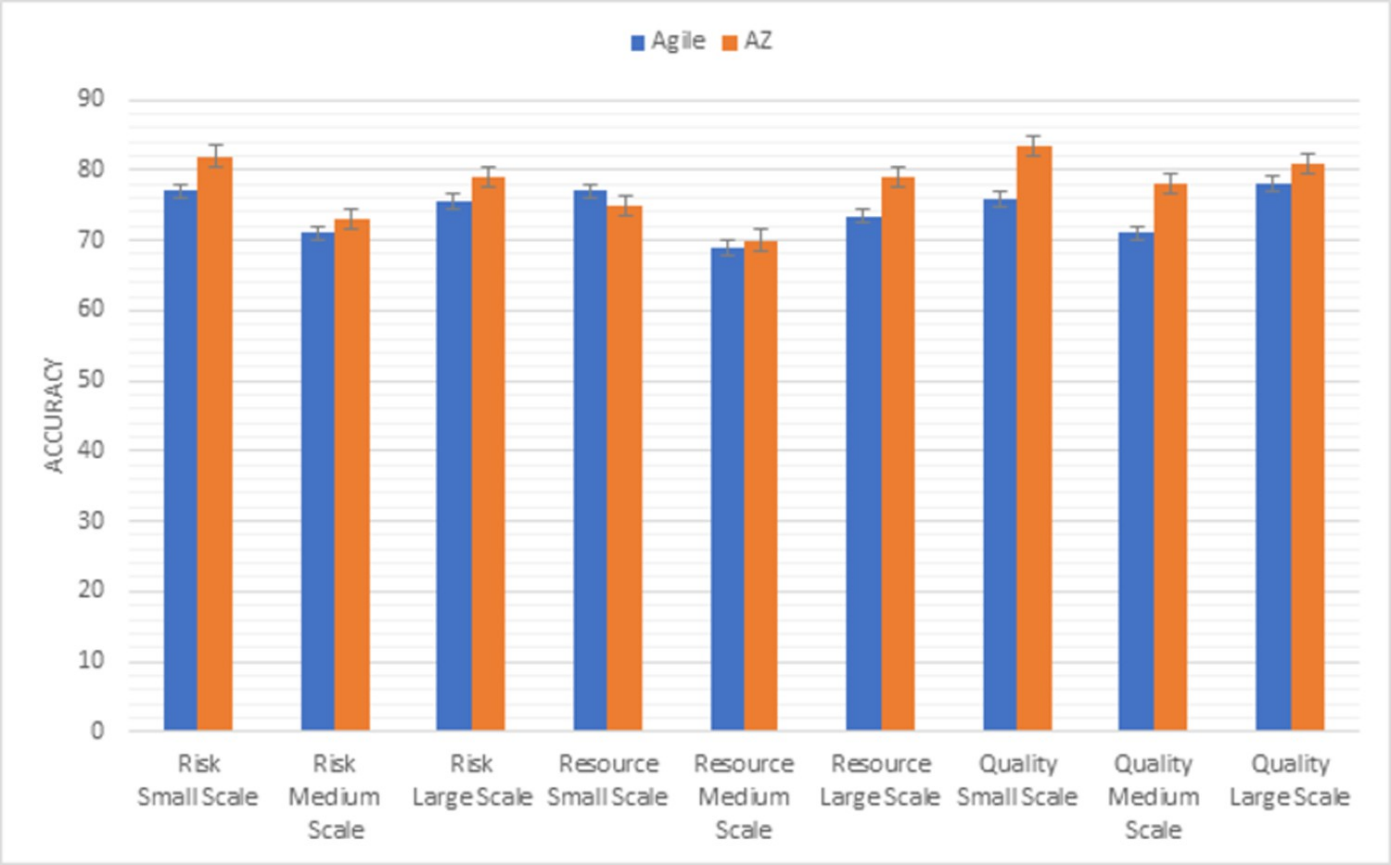
Graphical representation of agile vs AZ (second triangle) results.

Whereas, Fig 6 shows the results of the second triangle of this methodology factors (risk, resource, quality). The risk is handled in a better way in the AZ model as compared to the agile model in all sizes of projects. Maximum resources are used in the agile model in small-scale projects. In medium-scale projects, both models use equal resources but in large-scale projects, the AZ model is more preferable to achieve the desired product. Both models delivered good quality products but according to results, the AZ model is preferable to the agile model with a quality perspective because the main focus of the AZ model is to improve the quality of the product.

### Heavyweight vs heavyweight

#### Waterfall vs iterative

**Fig 7:**
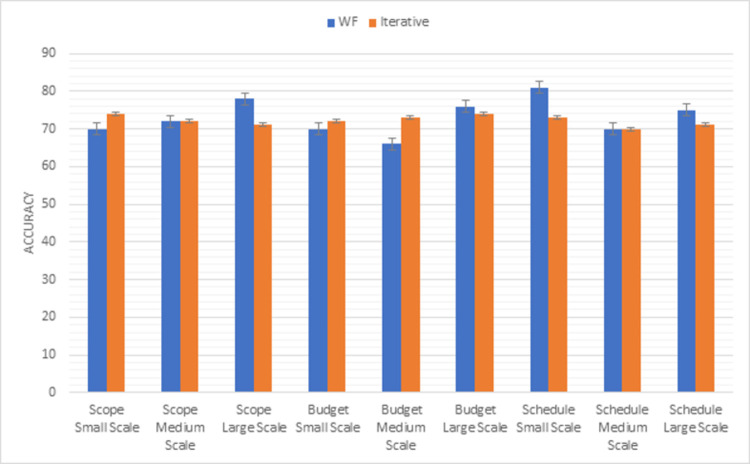
Graphical representation of waterfall vs iterative (first triangle) results.

The first process model is a waterfall model. It is a very simple model with some drawbacks. PMBOK methodology applies to these two models. Comparison results of the waterfall model with the iterative model using the first triangle of PMBOK methodology factors (scope, budget, schedule) are shown in Fig 7. In scope factor, the iterative model is more reliable than the waterfall model in small- and large-scale projects. For medium-scale projects, both models have the same results. According to the results of a budget factor the project accomplished within the decided budget is good in the iterative model than the waterfall model in small and medium scale projects but in large scale projects waterfall model is more reliable than the iterative model. In schedule factor, the waterfall model is more suitable for small-scale and medium-scale projects but for large-scale projects iterative model is best.

**Fig 8:**
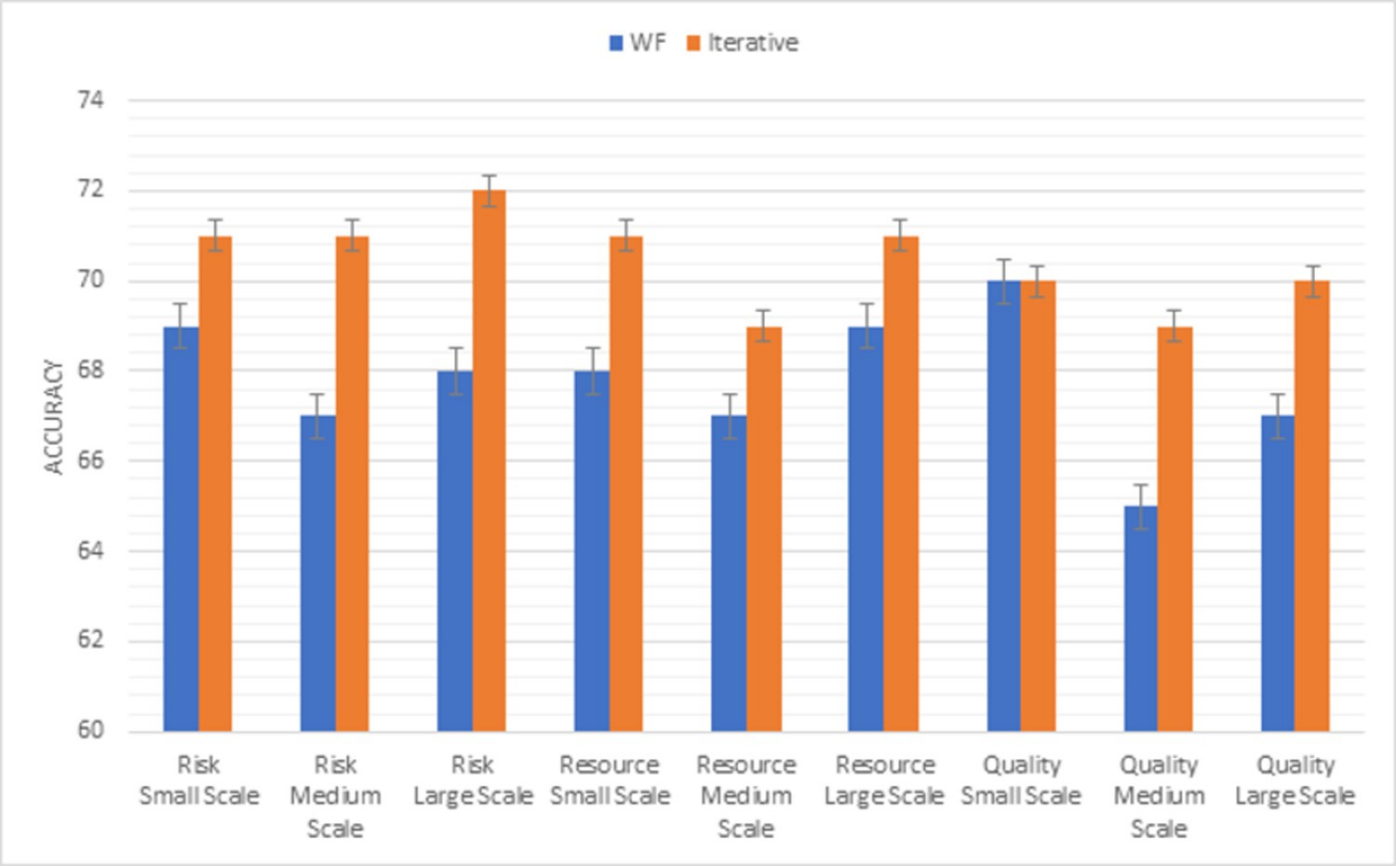
Graphical representation of waterfall vs iterative (second triangle) results.

Whereas Fig 8 shows the results of the second triangle of PMBOK methodology factors (risk, resource, quality). Risk is well managed in the iterative model in small medium and large-scale projects. Maximum resources are used in the iterative model in all types of projects. The most important factor is the quality factor. The iterative model provides a better-quality product than the waterfall model in medium and large-scale projects.

### Waterfall vs V

**Fig 9:**
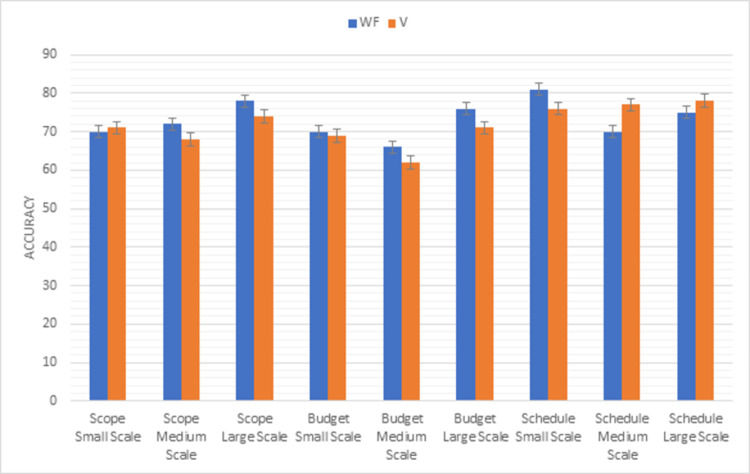
Graphical representation of waterfall vs V (first triangle) results.

**Fig 10:**
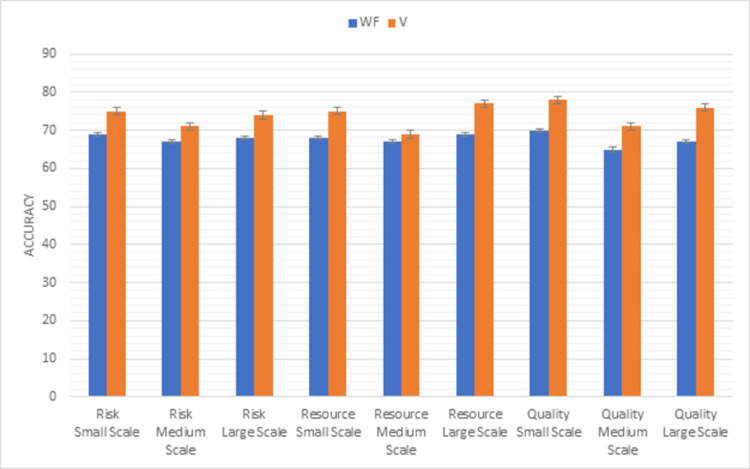
Graphical representation of waterfall vs V (second triangle) results.

Comparison results of waterfall model with V model using the first triangle of PMBOK methodology factors (scope, budget, schedule) are shown in Fig 9. In scope factor, the V model is more reliable in the small-scale project than the waterfall but in medium and large-scale projects waterfall model is preferable to the V model. The ratio of the project accomplished within the decided budget is higher in the waterfall model as compared to the V model in all types of projects. Scheduling of V model in small scale projects is better than waterfall model but in medium and large-scale projects waterfall model more preferable than V model,

Whereas, Fig 10 shows the results of the second triangle of this methodology factors (risk, resource, quality). In the V model risk can be minimized easily due to testing of every module in all sizes of projects. Maximum resource used in V model as compared to the waterfall model in all sizes of projects. The product developed by V-model is more efficient than the waterfall model in small medium and large size of projects.

### Iterative vs V

**Fig 11:**
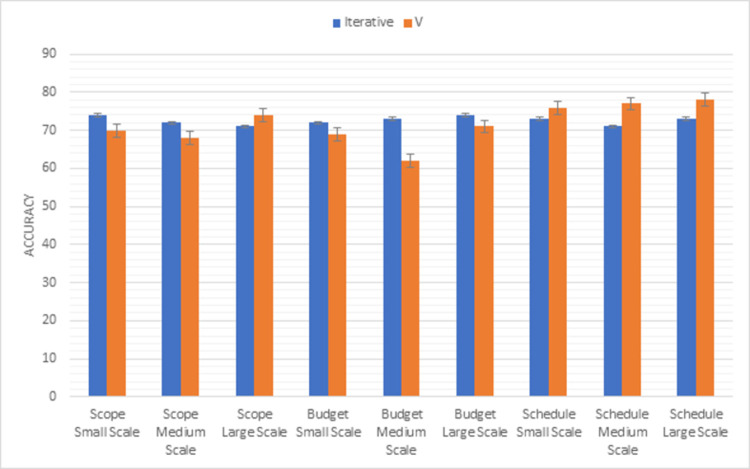
Graphical representation of iterative vs V (first triangle) results.

Comparison results of the Iterative model with the V model using the first triangle of PMBOK methodology factors (scope, budget, schedule) are shown in Fig 11. The scope factor of the iterative model is clearer than the V model in small and medium scale projects but in large-scale projects, the V model is more suitable than the iterative model. The iterative model performs all tasks within the decided budgets as compared to the V model in all sizes of projects. The scheduling factor gives the best result in the V model as compared to the Iterative model.

**Fig 12:**
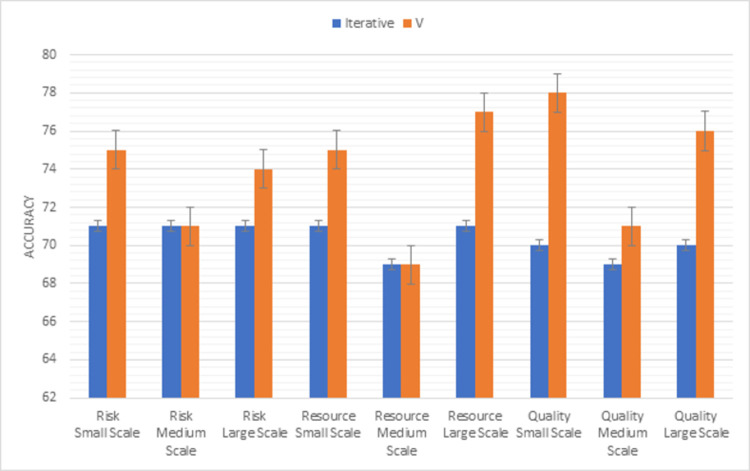
Graphical representation of iterative vs V (second triangle) results.

Whereas, Fig 12 shows the results of the second triangle of this methodology factors (risk, resource, quality). V model handles the risk in a well-managed way in all sizes of projects. Maximum resources are used in the V model to accomplish the task. When we compare the quality factor of both models, the V model is more efficient than the iterative model for all sizes of projects as compared to the iterative model.

**Fig 13:**
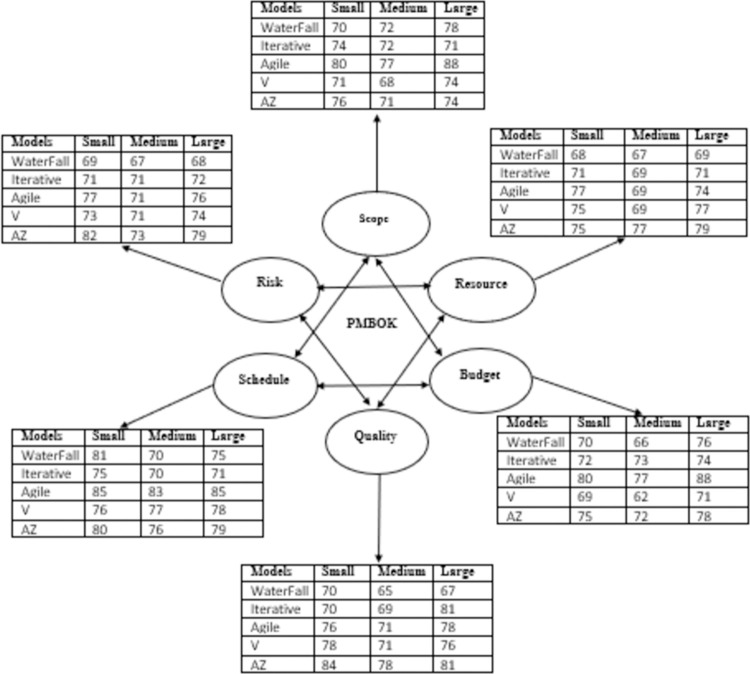
Result summary of different development models using PMBOK methodology.

The complete results summary based on a survey is demonstrated in Fig 13. In this plot, there is a comparative analysis of all development models based on the methodology of PMBOK which tells us that how all factors of the PMBOK model works in lightweight as well as heavyweight methodology. According to the result, the agile model is the most preferable in both methodologies. After the agile model, respondents focus on the AZ model which is also a modern technique for developing high-quality software. After the AZ model, the third priority of respondents is the V model. After the V model, Iterative and waterfall models are selected by respondents.

## Conclusion

The analyses were conducted to determine the best methodology according to the project size and the requirements of an organization. Software quality mainly depends on the selected software development model. In this research, there is a comparison of different software development models based on the PMBOK model to ensure the quality of projects. Based on the factors of the six-pointed star model the summarized results confirmed that almost all the factors of methodology are in favor of lightweight methodologies for small-scale projects. For medium-scale projects, both methodologies are almost similar. The concluded results of both methodologies for large-scale projects show that heavyweight methodologies are much more satisfactory for all factors of the six-pointed star model. The respondents prefer the agile model because agile is a customer-friendly model. The latest model is known as the AZ model which is suitable for small-scale and large-scale projects but customer interaction is also limited in this model which is the main disadvantage of this model but this model covers the drawback of all earlier models so this model is more reliable. There is a need to develop a new software model that is client-friendly also easy to use for developers with minimizing risk within decided budget and quality. In the future, we explore how intelligent software development models are based on a data-driven approach.

## Supporting information

S1 Dataset (XLSX)
