# Supplementary material for: Statistical analysis of software development models by six-pointed star framework
Source: PLoS One. 2022 Apr 1;17(4):e0264420. doi: 10.1371/journal.pone.0264420 (PMC8975137; doi:10.1371/journal.pone.0264420)
Supplement: Attachment — Submitted filename: Responses to reviewers.docx [file pone.0264420.s002.docx]

**Response Letter**

**List of Changes / Rebuttal**

Paper ID: PONE-D-21-05435	 	Status: **Revision**

Paper Title: Statistical Analysis of Software Development Models and Six-Pointed Star Methodology

Dear Editor,

Every desirable and necessary changes have been incorporated in the revised version. The authors owe gratitude to the referees for their valuable comments and suggestions which have helped us to improve the presentation of this paper. Please find below the rebuttal of our paper.

**Academic editor requirements:**

**Additional Requirement 1:**

1. Please ensure that your manuscript meets PLOS ONE's style requirements, including those for file naming. The PLOS ONE style templates can be found at https://journals.plos.org/plosone/s/file?id=wjVg/PLOSOne_formatting_sample_main_body.pdf and https://journals.plos.org/plosone/s/file?id=ba62/PLOSOne_formatting_sample_title_authors_affiliations.pdf

**Author response:** We ensure that we meet the requirements of PLOS ONE styles.

**Author action:** We have updated the manuscript according to PLOS ONE style.

**Additional Requirement** 2:

2. We suggest you thoroughly copyedit your manuscript for language usage, spelling, and grammar. If you do not know anyone who can help you do this, you may wish to consider employing a professional scientific editing service.  

Upon resubmission, please provide the following:

- The name of the colleague or the details of the professional service that edited your manuscript
- A copy of your manuscript showing your changes by either highlighting them or using track changes (uploaded as a *supporting information* file)
- A clean copy of the edited manuscript (uploaded as the new *manuscript* file)

**Author response:** Authors Intakhab Alam, Nadeem Sarwar, and Iram Noreen have edited the manuscript. The highlighting portion is the part that is changed/ updated by the authors and English Proof reader Ms. Robila Nosheen, Lecturer English Writing and Communication at Sabgha College of Masqat, Emirates. The certificate file duly signed by Ms. Robila is also uploaded in supplementary file section.

**Author action:** We have updated the manuscript file according to requirements. The spelling and grammar certificate file duly signed by proof reader Ms. Robila is also uploaded in supplementary file section.

**Additional Requirement** 3

Thank you for including your ethics statement:  "Ethical concerns mentioned in the article.
"To prevent the association of any ethical concerns to this particular research project,
certain measures were taken i.e. the research participants were asked about their
consent before participating in this research study. Also, no personal details of the
research participants were collected other than their email IDs, and the research
participants were informed about this act. All in all, the researcher practiced a high
level of morality and ethicality to attain and maintain the confidence of the research
participants. ".   Please amend your current ethics statement to include the full name of the ethics committee/institutional review board(s) that approved your specific study. Once you have amended this/these statement(s) in the Methods section of the manuscript, please add the same text to the “Ethics Statement” field of the submission form (via “Edit Submission”). For additional information about PLOS ONE ethical requirements for human subjects research, please refer to http://journals.plos.org/plosone/s/submission-guidelines#loc-human-subjects-research.

**Author response:** A post graduate research project evaluation and ethics committee has approved the study. The committee constitutes of 3 members, Dr. Ghulam Mustfa (Senior Assistant Professor), Dr. Abdul Hafeez (Associate Professor) and Dr. Qasim Khawaja (Head of Department Compyter Science). The committee approved the ethical review form of the study stated as FORM MS-9. The MS-9 ethical review form is also part of the post graduate thesis evaluation SOPs in the department of Computer Science, Bahria Unviersity, Pakistan.

**Author action :**

A MS-9 ethical review form is included in the Appendix-A. Moreover, an ethics statement is also added including ethical review board committee members names and designation in the mansuciript Section Ethics statement. Similarly ethics aspects approval by ethocs review committee is also described in Methodology Section.

**Additional Requirement** 4

4. Please provide additional details regarding participant consent. In the ethics statement in the Methods and online submission information, please ensure that you have specified what type you obtained (for instance, written or verbal, and if verbal, how it was documented and witnessed). If your study included minors, state whether you obtained consent from parents or guardians. If the need for consent was waived by the ethics committee, please include this information.

**Author response:** In the questionnaire form there is a statement related to informed consent in which we have ensured the respondent that their information will be kept confidential and will be used only for research purposes. Further, we have collected only respondent’s names and email ids. If respondent willing to provide their all information

**Author action:** The required action is already performed by discussion of informed consent in the Ethical concern section on page 10.

**Additional Requirement** 5

5. In your Data Availability statement, you have not specified where the minimal data set underlying the results described in your manuscript can be found. PLOS defines a study's minimal data set as the underlying data used to reach the conclusions drawn in the manuscript and any additional data required to replicate the reported study findings in their entirety. All PLOS journals require that the minimal data set be made fully available. For more information about our data policy, please see <http://journals.plos.org/plosone/s/data-availability>. Upon re-submitting your revised manuscript, please upload your study’s minimal underlying data set as either Supporting Information files or to a stable, public repository and include the relevant URLs, DOIs, or accession numbers within your revised cover letter. For a list of acceptable repositories, please see http://journals.plos.org/plosone/s/data-availability#loc-recommended-repositories. Any potentially identifying patient information must be fully anonymized.

Important: If there are ethical or legal restrictions to sharing your data publicly, please explain these restrictions in detail. Please see our guidelines for more information on what we consider unacceptable restrictions to publicly sharing data: http://journals.plos.org/plosone/s/data-availability#loc-unacceptable-data-access-restrictions. Note that it is not acceptable for the authors to be the sole named individuals responsible for ensuring data access.

We will update your Data Availability statement to reflect the information you provide in your cover letter.

**Author response:** The result is based on the minimal underlying dataset which is uploaded in a supporting information file in supplementary file section and is explained in the data collection section.

**Author action:** We have updated the manuscript and have uploaded minimal dataset in supporting file and have also discussed it in data collection section page no 9.

**Additional Requirement** 6

6. Please include your tables as part of your main manuscript and remove the individual files. Please note that supplementary tables should be uploaded as separate "supporting information" files.

**Author response:** All tables are included in the manuscript and a supplementary file of tables is also uploaded.

**Author action:** We have updated the manuscript according to requirements.

**Additional Requirement** 7

7. Please include captions for *all* your Supporting Information files at the end of your manuscript, and update any in-text citations to match accordingly. Please see our Supporting Information guidelines for more information: http://journals.plos.org/plosone/s/supporting-information.

**Author response:** We have updated the manuscript file and supporting information is also added at the end of the file.

**Author action:** We have updated the manuscript according to requirements.

**Reviewer 1 concerns:**

**Concern 1**

Reviewer #1: The authors compare several software development models by administering a survey to several software development companies. The survey asks respondents to indicate their preferred model and then proceeds to ask them several questions about their experiences in small, medium, and large software development projects in both lightweight and heavy methodologies. Based on this the authors analyze the pros/cons of the reported software models.

**Author response:** The authors have examined the different software development models and have designed a questionnaire based on PMBOK methodology known as the six-pointed star model. Then the author have acquired the opinion of experts of different methodologies of small medium and large-scale projects. Based on the opinion of experts author have finalized the results. Pros and cons are also discussed in a table as discussed in reviewer’s concern No 5.

**Author action:** No updates required in the manuscript.

**Concern 2**

While the objective of comparing several software models is a good one, the manuscript is not. It is written like a presentation that has been converted into a word document. Most of it consists almost entirely of bulleted lists with very few details and references to supporting evidence. There is a related works table but it is presented as is, without any elaboration. Figures have extremely short captions without descriptions of their visual elements. Proper statistical analyses are nonexistent and the language is poor.

**Author response:** The manuscript is updated by removing the bullet list, tables and figures are properly captioned.

**Author action:** We have updated the manuscript by explaining the concept on page 4 as required by the reviewer.

**Concern 3**

Regarding the survey, it asks a participant their opinion on two methodologies: lightweight and heavy. What if the participant is only experienced in one of these methodologies? Also, what if the participant indicates that they use more than one model in their organization? these situations are not explained in the text.

**Author response:** Responses were collected from experienced members of different organizations who know the software methodologies briefly. And if there is an organization that uses more than one model, respondents fill two forms.

**Author action:** We have updated the manuscript by explaining the concept on pages 4 and 8 respectively as required by the reviewer.

**Concern 4**

- Fixing spelling and grammar mistakes.

**Author response:** All the grammar and spelling mistakes are fixed by using Grammarly. Mansucript is thoroughly proff read by an english languga expert.

**Author action:** We have updated the manuscript as required.

**Concern 5**

- Unification of the pros and cons of various methodologies, as reported by literature, so that they could be summarized into a nice table and discussed afterward.

- Adding detailed discussion of related works.
- Replacing bulleted lists with text, including extra details on the points mentioned in those lists (along with proper citations).

**Author response:** The pros and cons table is included in the manuscript and bullets are replaced with the text form.

**Author action:** We have updated the manuscript section software development models on pages 4 to 6 with desired modifications suggested by honorable reviewer.

**Concern 6**

- Describing the visual elements of the figures properly.
- Performing and reporting proper statistical analysis (e.g., hypothesis testing, Bayesian approaches, etc).
- Reporting the results about the background of survey respondents (section A of the survey).
- Adding error bars to the figures.
- Ditching 3D visualizations (Fig 9) and using simpler more intuitive 2D histograms.

**Author response:** The visual elements of figures are properly described in revised manuscript and the background of the respondents is also mentioned in the data collection section. Error bar is also provided in the figures. Fig are updated using simpler or more intuitive 2d histogram.

**Author action:** We have updated the manuscript data collection section, result section, by explaining the concept on page 9 as suggested by reviewer.

**Concern 7**

Also, the authors should consider objective measures such as the organization's ability to deliver projects on time, budget, and scope. Objective measures are nice because they are not affected by subjective biases inherent in survey-based methods. Also, it might be useful to understand what kind of products the companies develop, as different methodologies might work best for different product types, not just (large, small, medium scale) products.

**Author response:** Different methodologies are used for different products of categories such as small-medium and large scale projects. Characteristics of different methodologies show which methodology is for small-medium or large-scale projects (page 5).

**Author action:** Characteristics of all methodologies are already discussed in the manuscript therefore, this concern requires not update.

**Reviewer 2 concerns:**

**Concern 1**

1. The manuscript gives an impression of a short presentation with separate slides. The description is weak and declarative. All text of the model comparison can be summarized in one table but then there is no text of the manuscript at all. Just an indication is “see the table”.

**Author response**: In the manuscript table presents the summarized form of the research work. However, we have added the text for table description to improve the manuscript as suggested by reviewer.

**Author action:** We have updated the manuscript methodology section by explaining the concepts on page 6.

**Concern 2**

2. The illustrations in Figures 2,3,4 are too obvious. Moreover, they are the same as in the paper by Akbar et. al (Akbar, M. A., Sang, J., Khan, A. A., Fazal, E. A., Nasrullah, Shafiq, M., Hussain, S., Hu, H., Elahi, M., and Xiang, H. (2017) Improving the quality of software development process by introducing a new methodology-Az-model. IEEE Access 6, 4811-4823.) The authors make reference number 19 to this paper but there is no indication of it in the Figure legend. Thus, the question of the originality of the drawings immediately arises. Additionally, even in an original publication by Akbar et. al a Six-pointed star model (Fig.5) is taken from free sources: wikipedia.org/wiki/File: TripleConstraint.jpg.

**Author response: F**igures 2,3,4 are the methodology figures named six-pointed star methodology. These figures are adapted from Akbar et. al (Akbar, M. A., Sang, J., Khan, A. A., Fazal, E. A., Nasrullah, Shafiq, M., Hussain, S., Hu, H., Elahi, M., and Xiang, H. (2017) Improving the quality of software development process by introducing a new methodology-Az-model. IEEE Access 6, 4811-4823.) paper. We have properly cited these figures in the manuscript.

**Author action:** We have updated the manuscript methodology section by explaining the concept on page 8 and figures are also cited.

**Concern 3**

There is no description of the statistical analysis made at all. The authors just represent several very unclear diagrams where the results have been plotted tightly with nearly invisible box-whisker output. The figure legends are very short with no descriptions.

**Author response:** We have clearly stated all diagrams in the result sections. The figure legends are visible and described clearly.

**Author action:** We have update the manuscript result section by explaining the concept on page 10.

**Concern 4**

4. The conclusions are unconvinced. The main reason for this observation is a poor depiction of the method and results. The postulate (page 19):
“Some models suitable for small and some suitable for large-scale projects. Some models have poor and some have good client interaction.” is common and uninformative.

**Author response:** We have updated the results and conclusion section accordingly.

**Author action:** We have updated the manuscript conclusion section by explaining the concept on page 21.

**Concern 5**

5. A reader can make an opinion about the manuscript like a presentation of the business result but not analytic material. To improve this impression the authors have to re-write methods and results and represent the outcomes.

**Author response:** We have revised the result sections, diagrams, and outcomes or improved discussion.

**Author action:** We have updated the manuscript result section as well as the conclusion on pages 10 and 21.

**Concern 6**

The form of the manuscript edition is poor as well. For example, reference 13 has no appropriate authors’ list. Additionally, the text must be edited by English native speakers.

**Author response:** We have added references with the help of the Mendeley tool.

**Author actions:** We update the manuscript reference 13 on page 22and similarly all references are checked for missing information entries using MS word insert citation.

**Once again, authors are grateful to all reviewers for their valuable time and suggestions to improve the mansucript.**
