# Supplementary material for: Statistical analysis of software development models by six-pointed star framework
Source: PLoS One. 2022 Apr 1;17(4):e0264420. doi: 10.1371/journal.pone.0264420 (PMC8975137; doi:10.1371/journal.pone.0264420)
Supplement: Attachment — Submitted filename: Responses to reviewers v 1.2.docx [file pone.0264420.s003.docx]

**Response Letter**

**List of Changes / Rebuttal**

Paper ID: PONE-D-21-05435R1	 	Status: **Revision**

Paper Title: Statistical Analysis of Software Development Models and Six-Pointed Star Methodology

Dear Editor,

Every desirable and necessary changes have been incorporated in the revised version. The authors owe gratitude to the referees for their valuable comments and suggestions which have helped us to improve the presentation of this paper. Please find below the rebuttal of our paper.

**Concern 1:**: The background section is still poor. First, it starts at too high a level by trying to convince us why computers are important. Second, papers are discussed briefly and simply placed in a table format, one sentence each, which is not enough to understand how this work is different from other papers which have attempted to compare software models and methodologies.

**Author response:** Back ground description in literature review section is revised according to reviewer’s suggestion.

**Author actions:** Back ground description in literature review section is revised according to reviewer’s suggestion. Bulleted lists and irrelevant headings are removed. First para about computers introduction is also removed. Remaining paragraphs are also revised/rephrased and proof read. Difference of previous study with this study is also explained on page 6 after Table 2.

**Concern 2:** There is almost zero discussion of each results Figure. The manuscript does not tell the readers what the results imply in each plot. Because of this and the lack of y-axis labels in the plots, it is unclear how the authors reached their conclusions regarding the efficacy of different models.

**Author response:** We have updated the results figures discussion section and also plot y-axis labels properly. The resulting figure clearly shows that the comparison of small medium and large-scale projects concerning adopted methodology.

**Author actions:** We update the results figure section with a detailed discussion also update y-axis labels.

**Concern 3:** Does the distinction between "methodology" and "model" imply that the two are orthogonal? For example, can you have a heavyweight agile combination? or a lightweight waterfall combination? it certainly appears like the two are NOT orthogonal. If that's the case, what's the point of this distinction?

**Author response:** A model provides an environment to implement a framework. A methodology is a tool that helps to perform some action. In our research work, our adopted methodology factors judge different software development models based on heavyweight as well as lightweight by factor wise for example budget factor (agile vs waterfall) budget analyze of both models in light as well as heavyweight in a sequential manner.

**Author actions:** We compare two models with respect to Small Medium and large scale projects also evaluate these models in lightweight as well as heavyweight methodologies.

**Concern 4:** It seems unreasonable to me to expect that participants in small organizations can have strong experience with large-scale projects, and vice-versa. How do the authors control for this in their statistical analysis? and how do they control for the respondent's programming experience?

**Author response**: In the data collection method, we explain our respondents have experienced people even in organizations small or large. Respondent programming experience control in questionnaire form.

**Author actions:** we update the data collection method on page 10.

**Concern 5:** What is the distribution of software models that the respondents have selected (i.e., how many selected waterfalls, AZ, agile, etc)?

- Plots are still missing y-axis labels.

- Error bars appear to be identical in length for same-colored bars in each plot. Why is that?

**Author response:** We add the distribution of software models that the respondents have selected in the form of a table in the supporting file also plot the y-axis labels.

**Author actions:** We update the data collection method section on page 10 and also update the result section figure on page 22.

**Concern 6:** To summarize their results, I suggest using a star diagram similar to Figure 2, to show where each model's strengths lie.

**Author response**: We use a star diagram summarize the result results adequately.

**Author actions:** we update the resulting figure on page 22.

**Concern 7:** The authors should re-examine their manuscript's language. There are clear typos, grammatical errors, and weird phraseology everywhere. A few examples:
- "After completing the paling phase ..." (page 3) -> After completing the planning phase ...
- " the iterative model adopts which is mainly focus on the coding and testing phase" (page 5) -> the iterative model focuses mainly on coding and testing phases
- "Now in the modern year," (page 8)
- "Questioner related to schedule" (Table 4) -> "Questions related to schedule"

**Author response**: Manuscript is revised to improve writing and typos.

**Author actions:** Manuscript is revised to improve writing and typos.

**Concern 8:** Software development models are still unnecessarily listed in a bulleted list in page 6.

**Author response**: Bullets are removed as advised.

**Author actions:** Bullets are removed as advised.

**Concern 9:** Data availability: the authors include summary data (Table 6) in the supplementary file but it is unclear what the numbers in the table mean. Proper data availability for this manuscript means that the authors must include all survey results (taking care to anonymize the authors and organizations of course).

**Author response**: We have update the required table descriptions.

**Author actions:** we update the description about table 6 and as well as table 7 on page 12.

**Once again, authors are grateful to all reviewers for their valuable time and suggestions to improve the manuscript.**
