# Supplementary material for: Statistical analysis of software development models by six-pointed star framework
Source: PLoS One. 2022 Apr 1;17(4):e0264420. doi: 10.1371/journal.pone.0264420 (PMC8975137; doi:10.1371/journal.pone.0264420)
Supplement: Attachment — Submitted filename: Responses to reviewers v 1.3.docx [file pone.0264420.s004.docx]

**Response Letter**

**List of Changes / Rebuttal**

Paper ID: PONE-D-21-05435R2	 	Status: **Revision**

Paper Title: Statistical Analysis of Software Development Models and Six-Pointed Star Methodology

Dear Editor,

The authors owe gratitude to the referees for their valuable comments and suggestions which have helped us to improve the presentation of this paper. Every desirable and necessary changes have been incorporated in the revised version. Please find below the rebuttal of our paper.

**Concern** **1**:

First, the authors make an artificial distinction between a “methodology” and a “model”. The way they define it, one can have a “heavyweight agile” combination or a “lightweight waterfall” combination. None of these combinations make any sense; an agile methodology is lightweight. This problematic distinction exists in the survey used to gather the programmers’ feedback. Specifically, the survey asks respondents to indicate the software methodology followed in their company (agile, waterfall, etc), then proceeds to ask them a series of questions, each requiring them to differentiate between a “lightweight” and “heavyweight” methodology. As stated previously, this survey structure means that one can have heavy agile or light waterfall methods, which is nonsensical. The authors’ abbreviated response to this concern was not satisfactory. It would have been better to group the software development models into the two categories instead. For example, the agile model belongs to the “lightweight” category, while the waterfall model belongs to the “heavyweight” category.

**Author response**: A model is templete while methodology is study of method that is used in a field.In the updated manuscript the lightweight and heavyweight methodologies are discussed separately. The comparative analysis of lightweight vs lightweight and heavyweight vs heavyweight are discussed separately. The waterfall model belongs to heavyweight methodology so its comparison is done only with the models that belong to heavyweight methodology. The lightweight methodology (agile, AZ) comparison is done within lightweight boundaries. For the categorization of small medium and large scale projects, there is table 7 added to the manuscript.

**Author actions:** We have updated the manuscript.

**Concern** **2**:

Second, the survey assumes that all programmers have experience with both “heavyweight” and “lightweight” methodologies. Again, the authors response to this concern was not satisfactory. They stated that all respondents had 3-5 years’ experience. But that doesn’t mean that all of them have experience with small- and large-scale projects or lightweight and heavyweight methodologies. For example, programmers might be highly experienced but work within small development shops which might favour lighter methods, and vice versa. The survey only asks how many years’ experience the respondent has, not the size of projects they have been involved in the past. This casts serious doubt on the results of the survey as it is highly likely that respondents are only responding with what they perceive to be the advantages and disadvantages of lightweight and heavyweight methodologies, rather than what the respondents experienced when following those methodologies.

**Author response:** For the categorization of small medium and large scale projects, there is table 7 added to the manuscript. In the updated survey questionnaire form the experience section is divided into further subsections (lightweight and heavyweight or both) respondent give their opinion about experiences in lightweight and heavyweight methodologies. The questions relating to scope and risk in the survey are revised. Survey Questioner updated according to reviewer recommendation

**Author actions:** We have updated the manuscript.

**Concern** **3**:

Third, it seems to me that some aspects of the 6-star evaluation methodology can be better served by using objective measures rather than surveys. Scheduling, budget, and resources can be measured objectively in terms of time, money, and capital spent. Quality can be measured in terms of unit-test coverage, number of bugs, post-release issues, cyclomatic complexity, etc.

**Author response:** In 6-star evaluation methodology (Scheduling, budget, and resources) are measured in term of time money and capital but quality measured in term of customer satisfaction and success of project.

**Author actions:** We have updated the manuscript.

**Concern** **4**:

Fourth, the questions relating to scope and risk in the survey are very vague. For example, in the scope section there is this question: “Does the project have a decisive scope by adopting a lightweight methodology”. What does decisive scope mean? How can the respondent know? Similarly, what is meant by meeting business aspirations under the risk section?

**Author response:** The questions relating to scope and risk in the survey are revised. Survey Questioner updated according to reviewer recommendation

**Author actions:** We have updated the manuscript.
