# Supplementary material for: Statistical analysis of software development models by six-pointed star framework
Source: PLoS One. 2022 Apr 1;17(4):e0264420. doi: 10.1371/journal.pone.0264420 (PMC8975137; doi:10.1371/journal.pone.0264420)
Supplement: Peer review history

# Peer review: Decision Letter 0

**DOI:** 10.1371/journal.pone.0264420.r001

**Authors:** M. Usman Ashraf

17 May 2021

PONE-D-21-05435

Statistical Analysis of Software Development Models and Six-Pointed Star Methodology

PLOS ONE

Dear Dr. SARWAR,

Thank you for submitting your manuscript to PLOS ONE. After careful consideration, we feel that it has merit but does not fully meet PLOS ONE’s publication criteria as it currently stands. Therefore, we invite you to submit a revised version of the manuscript that addresses the points raised during the review process.

Please submit your revised manuscript by Jul 01 2021 11:59PM. If you will need more time than this to complete your revisions, please reply to this message or contact the journal office at plosone@plos.org. When you're ready to submit your revision, log on to https://www.editorialmanager.com/pone/ and select the 'Submissions Needing Revision' folder to locate your manuscript file.

Please include the following items when submitting your revised manuscript:

- A rebuttal letter that responds to each point raised by the academic editor and reviewer(s). You should upload this letter as a separate file labeled 'Response to Reviewers'.
- A marked-up copy of your manuscript that highlights changes made to the original version. You should upload this as a separate file labeled 'Revised Manuscript with Track Changes'.
- An unmarked version of your revised paper without tracked changes. You should upload this as a separate file labeled 'Manuscript'.

If you would like to make changes to your financial disclosure, please include your updated statement in your cover letter. Guidelines for resubmitting your figure files are available below the reviewer comments at the end of this letter.

If applicable, we recommend that you deposit your laboratory protocols in protocols.io to enhance the reproducibility of your results. Protocols.io assigns your protocol its own identifier (DOI) so that it can be cited independently in the future. For instructions see: http://journals.plos.org/plosone/s/submission-guidelines#loc-laboratory-protocols. Additionally, PLOS ONE offers an option for publishing peer-reviewed Lab Protocol articles, which describe protocols hosted on protocols.io. Read more information on sharing protocols at https://plos.org/protocols?utm_medium=editorial-email&utm_source=authorletters&utm_campaign=protocols.

We look forward to receiving your revised manuscript.

Kind regards,

M. Usman Ashraf, Ph.D

Academic Editor

PLOS ONE

Journal Requirements:

When submitting your revision, we need you to address these additional requirements.

1. Please ensure that your manuscript meets PLOS ONE's style requirements, including those for file naming. The PLOS ONE style templates can be found at

https://journals.plos.org/plosone/s/file?id=wjVg/PLOSOne_formatting_sample_main_body.pdf and

https://journals.plos.org/plosone/s/file?id=ba62/PLOSOne_formatting_sample_title_authors_affiliations.pdf

2. We suggest you thoroughly copyedit your manuscript for language usage, spelling, and grammar. If you do not know anyone who can help you do this, you may wish to consider employing a professional scientific editing service.  

Whilst you may use any professional scientific editing service of your choice, PLOS has partnered with both American Journal Experts (AJE) and Editage to provide discounted services to PLOS authors. Both organizations have experience helping authors meet PLOS guidelines and can provide language editing, translation, manuscript formatting, and figure formatting to ensure your manuscript meets our submission guidelines. To take advantage of our partnership with AJE, visit the AJE website (http://learn.aje.com/plos/) for a 15% discount off AJE services. To take advantage of our partnership with Editage, visit the Editage website (www.editage.com) and enter referral code PLOSEDIT for a 15% discount off Editage services.  If the PLOS editorial team finds any language issues in text that either AJE or Editage has edited, the service provider will re-edit the text for free.

Upon resubmission, please provide the following:

- The name of the colleague or the details of the professional service that edited your manuscript
- A copy of your manuscript showing your changes by either highlighting them or using track changes (uploaded as a *supporting information* file)
- A clean copy of the edited manuscript (uploaded as the new *manuscript* file)

3. Thank you for including your ethics statement:  "Ethical concerns mentioned in the article.

"To prevent the association of any ethical concerns to this particular research project,

certain measures were taken i.e. the research participants were asked about their

consent before participating in this research study. Also, no personal details of the

research participants were collected other than their email IDs, and the research

participants were informed about this act. All in all, the researcher practiced a high

level of morality and ethicality to attain and maintain the confidence of the research

participants."".   

Please amend your current ethics statement to include the full name of the ethics committee/institutional review board(s) that approved your specific study.

Once you have amended this/these statement(s) in the Methods section of the manuscript, please add the same text to the “Ethics Statement” field of the submission form (via “Edit Submission”).

For additional information about PLOS ONE ethical requirements for human subjects research, please refer to http://journals.plos.org/plosone/s/submission-guidelines#loc-human-subjects-research.

4. Please provide additional details regarding participant consent. In the ethics statement in the Methods and online submission information, please ensure that you have specified what type you obtained (for instance, written or verbal, and if verbal, how it was documented and witnessed). If your study included minors, state whether you obtained consent from parents or guardians. If the need for consent was waived by the ethics committee, please include this information.

5. In your Data Availability statement, you have not specified where the minimal data set underlying the results described in your manuscript can be found. PLOS defines a study's minimal data set as the underlying data used to reach the conclusions drawn in the manuscript and any additional data required to replicate the reported study findings in their entirety. All PLOS journals require that the minimal data set be made fully available. For more information about our data policy, please see http://journals.plos.org/plosone/s/data-availability.

Upon re-submitting your revised manuscript, please upload your study’s minimal underlying data set as either Supporting Information files or to a stable, public repository and include the relevant URLs, DOIs, or accession numbers within your revised cover letter. For a list of acceptable repositories, please see http://journals.plos.org/plosone/s/data-availability#loc-recommended-repositories. Any potentially identifying patient information must be fully anonymized.

Important: If there are ethical or legal restrictions to sharing your data publicly, please explain these restrictions in detail. Please see our guidelines for more information on what we consider unacceptable restrictions to publicly sharing data: http://journals.plos.org/plosone/s/data-availability#loc-unacceptable-data-access-restrictions. Note that it is not acceptable for the authors to be the sole named individuals responsible for ensuring data access.

We will update your Data Availability statement to reflect the information you provide in your cover letter.

6. Please include your tables as part of your main manuscript and remove the individual files. Please note that supplementary tables should be uploaded as separate "supporting information" files.

7. Please include captions for *all* your Supporting Information files at the end of your manuscript, and update any in-text citations to match accordingly. Please see our Supporting Information guidelines for more information: http://journals.plos.org/plosone/s/supporting-information.

[Note: HTML markup is below. Please do not edit.]

Reviewers' comments:

Reviewer's Responses to Questions

**Comments to the Author**

1. Is the manuscript technically sound, and do the data support the conclusions?

The manuscript must describe a technically sound piece of scientific research with data that supports the conclusions. Experiments must have been conducted rigorously, with appropriate controls, replication, and sample sizes. The conclusions must be drawn appropriately based on the data presented. 

Reviewer #1: No

Reviewer #2: Partly

**********

2. Has the statistical analysis been performed appropriately and rigorously? 

Reviewer #1: No

Reviewer #2: N/A

**********

3. Have the authors made all data underlying the findings in their manuscript fully available?

The PLOS Data policy requires authors to make all data underlying the findings described in their manuscript fully available without restriction, with rare exception (please refer to the Data Availability Statement in the manuscript PDF file). The data should be provided as part of the manuscript or its supporting information, or deposited to a public repository. For example, in addition to summary statistics, the data points behind means, medians and variance measures should be available. If there are restrictions on publicly sharing data—e.g. participant privacy or use of data from a third party—those must be specified.

Reviewer #1: No

Reviewer #2: No

**********

4. Is the manuscript presented in an intelligible fashion and written in standard English?

PLOS ONE does not copyedit accepted manuscripts, so the language in submitted articles must be clear, correct, and unambiguous. Any typographical or grammatical errors should be corrected at revision, so please note any specific errors here.

Reviewer #1: No

Reviewer #2: No

**********

5. Review Comments to the Author

Please use the space provided to explain your answers to the questions above. You may also include additional comments for the author, including concerns about dual publication, research ethics, or publication ethics. (Please upload your review as an attachment if it exceeds 20,000 characters)

Reviewer #1: The authors compare several software development models by administering a survey to several software development companies. The survey asks respondents to indicate their preferred model and then proceeds to ask them several questions about their experiences in small, medium, and large software development projects in both lightweight and heavy methodologies. Based on this the authors analyze the pros/cons of the reported software models.

While the objective of comparing several software models is a good one, the manuscript is not. It is written like a presentation that has been converted into a word document. Most of it consists almost entirely of bulleted lists with very little details and references to supporting evidence. There is a related works table but it is presented as is, without any elaboration. Figures have extremely short captions without descriptions of their visual elements. Proper statistical analyses are non existent and the language is poor.

Regarding the survey, it asks a participant their opinion on two methodologies: lightweight and heavy. What if the participant is only experienced in one of these methodologies? Also, what if the participant indicates that they use more than one model in their organization? these situations are not explained in the text.

As it is, this work is a definite rejection. To improve the manuscript, the authors should consider:

- Fixing spelling and grammar mistakes.

- Unification of the pros and cons of various methodologies, as reported by literature, so that they could be summarized into a nice table and discussed afterwords.

- Adding detailed discussion of related works.

- Replacing bulleted lists with text, including extra details on the points mentioned in those lists (along with proper citations).

- Describing the visual elements of the figures properly.

- Performing and reporting proper statistical analysis (e.g., hypothesis testing, Bayesian approaches, etc).

- Reporting the results about the background of survey respondents (section A of the survey).

- Adding error bars to the figures.

- Ditching 3D visualizations (Fig 9) and using simpler more intuitive 2D histograms.

Also, the authors should consider objective measures such as the organization's ability to deliver projects on time, budget, and scope. Objective measures are nice because they are not affected by subjective biases inherent in survey-based methods. Also, it might be useful to understand what kind of products the companies develop, as different methodologies might work best for different product types, not just (large, small, medium scale) products.

Reviewer #2: The submitted manuscript deals with the problem of different software development process models comparison to the six-pointed star methodology using a statistical analysis. Initially, the authors have considered six different models with arguments of their advantages and disadvantages. Then, the data of the survey with 31 responses is used for statistical analysis. Indeed, the main subject of the manuscript seems to be interesting. However, several failings must be indicated.

1. The manuscript gives an impression of a short presentation with the separate slides. The description is weak and declarative. All text of the model comparison can be summarized in a one table but then there is no text of the manuscript at all. Just an indication is “see the table”.

2. The illustrations in Figure 2,3,4 are too obvious. Moreover, they are exactly the same like in the paper by Akbar et. al (Akbar, M. A., Sang, J., Khan, A. A., Fazal, E. A., Nasrullah, Shafiq, M., Hussain, S., Hu, H., Elahi, M., and Xiang, H. (2017) Improving the quality of software development process by introducing a new methodology-Az-model. IEEE Access 6, 4811-4823.) The authors make a reference number 19 to this paper but there is no indication on it in the Figure legend. Thus, the question of originality of the drawings is immediately arisen. Additionally, even in an original publication by Akbar et. al a Six-pointed star model (Fig.5) is taken from free sources: wikipedia.org/wiki/File: TripleConstraint.jpg.

3. There is no description of the statistical analysis made at all. The authors just represent several very unclear diagrams where the results have been plotted tightly with nearly invisible box-whisker output. The figure legends are very short with no descriptions.

4. The conclusions are unconvinced. The main reason for this observation is a poor depiction of method and results. The postulate (page 19):

“Some models suitable for small and some suitable for large-scale projects. Some models have poor and some have good client interaction.”

is common and uninformative.

5. A reader can make an opinion about the manuscript like a presentation of business result but not an analytic material. To improve this impression the authors have to re-write methods and results and represent the outcomes clearly.

6. The form of the manuscript edition is poor as well. For example, the reference 13 has no appropriate authors’ list. Additionally, the text must be edited by English native speakers.

**********

6. PLOS authors have the option to publish the peer review history of their article (what does this mean?). If published, this will include your full peer review and any attached files.

If you choose “no”, your identity will remain anonymous but your review may still be made public.

**Do you want your identity to be public for this peer review?** For information about this choice, including consent withdrawal, please see our Privacy Policy.

Reviewer #1: No

Reviewer #2: No

[NOTE: If reviewer comments were submitted as an attachment file, they will be attached to this email and accessible via the submission site. Please log into your account, locate the manuscript record, and check for the action link "View Attachments". If this link does not appear, there are no attachment files.]

While revising your submission, please upload your figure files to the Preflight Analysis and Conversion Engine (PACE) digital diagnostic tool, https://pacev2.apexcovantage.com/. PACE helps ensure that figures meet PLOS requirements. To use PACE, you must first register as a user. Registration is free. Then, login and navigate to the UPLOAD tab, where you will find detailed instructions on how to use the tool. If you encounter any issues or have any questions when using PACE, please email PLOS at figures@plos.org. Please note that Supporting Information files do not need this step.

# Author comment: Author response to Decision Letter 0

**DOI:** 10.1371/journal.pone.0264420.r002

1 Jul 2021

Reviewer 1 concerns:

Concern 1

Reviewer #1: The authors compare several software development models by administering a survey to several software development companies. The survey asks respondents to indicate their preferred model and then proceeds to ask them several questions about their experiences in small, medium, and large software development projects in both lightweight and heavy methodologies. Based on this the authors analyze the pros/cons of the reported software models.

Author response: The authors have examined the different software development models and have designed a questionnaire based on PMBOK methodology known as the six-pointed star model. Then the author have acquired the opinion of experts of different methodologies of small medium and large-scale projects. Based on the opinion of experts author have finalized the results. Pros and cons are also discussed in a table as discussed in reviewer’s concern No 5.

Author action: No updates required in the manuscript.

Concern 2

While the objective of comparing several software models is a good one, the manuscript is not. It is written like a presentation that has been converted into a word document. Most of it consists almost entirely of bulleted lists with very few details and references to supporting evidence. There is a related works table but it is presented as is, without any elaboration. Figures have extremely short captions without descriptions of their visual elements. Proper statistical analyses are nonexistent and the language is poor.

Author response: The manuscript is updated by removing the bullet list, tables and figures are properly captioned.

Author action: We have updated the manuscript by explaining the concept on page 4 as required by the reviewer.

Concern 3

Regarding the survey, it asks a participant their opinion on two methodologies: lightweight and heavy. What if the participant is only experienced in one of these methodologies? Also, what if the participant indicates that they use more than one model in their organization? these situations are not explained in the text.

Author response: Responses were collected from experienced members of different organizations who know the software methodologies briefly. And if there is an organization that uses more than one model, respondents fill two forms.

Author action: We have updated the manuscript by explaining the concept on pages 4 and 8 respectively as required by the reviewer. 

Concern 4

- Fixing spelling and grammar mistakes.

Author response: All the grammar and spelling mistakes are fixed by using Grammarly. Mansucript is thoroughly proff read by an english languga expert.

Author action: We have updated the manuscript as required. 

Concern 5

- Unification of the pros and cons of various methodologies, as reported by literature, so that they could be summarized into a nice table and discussed afterward.

- Adding detailed discussion of related works.

- Replacing bulleted lists with text, including extra details on the points mentioned in those lists (along with proper citations).

Author response: The pros and cons table is included in the manuscript and bullets are replaced with the text form. 

Author action: We have updated the manuscript section software development models on pages 4 to 6 with desired modifications suggested by honorable reviewer.

Concern 6

- Describing the visual elements of the figures properly.

- Performing and reporting proper statistical analysis (e.g., hypothesis testing, Bayesian approaches, etc).

- Reporting the results about the background of survey respondents (section A of the survey).

- Adding error bars to the figures.

- Ditching 3D visualizations (Fig 9) and using simpler more intuitive 2D histograms.

Author response: The visual elements of figures are properly described in revised manuscript and the background of the respondents is also mentioned in the data collection section. Error bar is also provided in the figures. Fig are updated using simpler or more intuitive 2d histogram.

Author action: We have updated the manuscript data collection section, result section, by explaining the concept on page 9 as suggested by reviewer.

Concern 7

Also, the authors should consider objective measures such as the organization's ability to deliver projects on time, budget, and scope. Objective measures are nice because they are not affected by subjective biases inherent in survey-based methods. Also, it might be useful to understand what kind of products the companies develop, as different methodologies might work best for different product types, not just (large, small, medium scale) products.

Author response: Different methodologies are used for different products of categories such as small-medium and large scale projects. Characteristics of different methodologies show which methodology is for small-medium or large-scale projects (page 5).

Author action: Characteristics of all methodologies are already discussed in the manuscript therefore, this concern requires not update.

Reviewer 2 concerns:

Concern 1

1. The manuscript gives an impression of a short presentation with separate slides. The description is weak and declarative. All text of the model comparison can be summarized in one table but then there is no text of the manuscript at all. Just an indication is “see the table”.

Author response: In the manuscript table presents the summarized form of the research work. However, we have added the text for table description to improve the manuscript as suggested by reviewer.

Author action: We have updated the manuscript methodology section by explaining the concepts on page 6.

Concern 2

2. The illustrations in Figures 2,3,4 are too obvious. Moreover, they are the same as in the paper by Akbar et. al (Akbar, M. A., Sang, J., Khan, A. A., Fazal, E. A., Nasrullah, Shafiq, M., Hussain, S., Hu, H., Elahi, M., and Xiang, H. (2017) Improving the quality of software development process by introducing a new methodology-Az-model. IEEE Access 6, 4811-4823.) The authors make reference number 19 to this paper but there is no indication of it in the Figure legend. Thus, the question of the originality of the drawings immediately arises. Additionally, even in an original publication by Akbar et. al a Six-pointed star model (Fig.5) is taken from free sources: wikipedia.org/wiki/File: TripleConstraint.jpg.

Author response: Figures 2,3,4 are the methodology figures named six-pointed star methodology. These figures are adapted from Akbar et. al (Akbar, M. A., Sang, J., Khan, A. A., Fazal, E. A., Nasrullah, Shafiq, M., Hussain, S., Hu, H., Elahi, M., and Xiang, H. (2017) Improving the quality of software development process by introducing a new methodology-Az-model. IEEE Access 6, 4811-4823.) paper. We have properly cited these figures in the manuscript.

Author action: We have updated the manuscript methodology section by explaining the concept on page 8 and figures are also cited.

Concern 3

There is no description of the statistical analysis made at all. The authors just represent several very unclear diagrams where the results have been plotted tightly with nearly invisible box-whisker output. The figure legends are very short with no descriptions.

Author response: We have clearly stated all diagrams in the result sections. The figure legends are visible and described clearly.

Author action: We have update the manuscript result section by explaining the concept on page 10.

 

Concern 4

4. The conclusions are unconvinced. The main reason for this observation is a poor depiction of the method and results. The postulate (page 19):

“Some models suitable for small and some suitable for large-scale projects. Some models have poor and some have good client interaction.” is common and uninformative.

Author response: We have updated the results and conclusion section accordingly.

Author action: We have updated the manuscript conclusion section by explaining the concept on page 21.

Concern 5

5. A reader can make an opinion about the manuscript like a presentation of the business result but not analytic material. To improve this impression the authors have to re-write methods and results and represent the outcomes.

Author response: We have revised the result sections, diagrams, and outcomes or improved discussion. 

Author action: We have updated the manuscript result section as well as the conclusion on pages 10 and 21.

Concern 6

The form of the manuscript edition is poor as well. For example, reference 13 has no appropriate authors’ list. Additionally, the text must be edited by English native speakers.

Author response: We have added references with the help of the Mendeley tool. 

Author actions: We update the manuscript reference 13 on page 22and similarly all references are checked for missing information entries using MS word insert citation.

Once again, authors are grateful to all reviewers for their valuable time and suggestions to improve the mansucript.

Attachment Submitted filename: Responses to reviewers.docx Click here for additional data file.

# Peer review: Decision Letter 1

**DOI:** 10.1371/journal.pone.0264420.r003

**Authors:** M. Usman Ashraf

26 Jul 2021

PONE-D-21-05435R1

Statistical Analysis of Software Development Models and Six-Pointed Star Methodology

PLOS ONE

Dear Dr. SARWAR,

Thank you for submitting your manuscript to PLOS ONE. After careful consideration, we feel that it has merit but does not fully meet PLOS ONE’s publication criteria as it currently stands. Therefore, we invite you to submit a revised version of the manuscript that addresses the points raised during the review process.

Please submit your revised manuscript by Sep 09 2021 11:59PM. If you will need more time than this to complete your revisions, please reply to this message or contact the journal office at plosone@plos.org. When you're ready to submit your revision, log on to https://www.editorialmanager.com/pone/ and select the 'Submissions Needing Revision' folder to locate your manuscript file.

Please include the following items when submitting your revised manuscript:

- A rebuttal letter that responds to each point raised by the academic editor and reviewer(s). You should upload this letter as a separate file labeled 'Response to Reviewers'.
- A marked-up copy of your manuscript that highlights changes made to the original version. You should upload this as a separate file labeled 'Revised Manuscript with Track Changes'.
- An unmarked version of your revised paper without tracked changes. You should upload this as a separate file labeled 'Manuscript'.

If you would like to make changes to your financial disclosure, please include your updated statement in your cover letter. Guidelines for resubmitting your figure files are available below the reviewer comments at the end of this letter.

If applicable, we recommend that you deposit your laboratory protocols in protocols.io to enhance the reproducibility of your results. Protocols.io assigns your protocol its own identifier (DOI) so that it can be cited independently in the future. For instructions see: http://journals.plos.org/plosone/s/submission-guidelines#loc-laboratory-protocols. Additionally, PLOS ONE offers an option for publishing peer-reviewed Lab Protocol articles, which describe protocols hosted on protocols.io. Read more information on sharing protocols at https://plos.org/protocols?utm_medium=editorial-email&utm_source=authorletters&utm_campaign=protocols.

We look forward to receiving your revised manuscript.

Kind regards,

M. Usman Ashraf, Ph.D

Academic Editor

PLOS ONE

Journal Requirements:

Additional Editor Comments (if provided):

[Note: HTML markup is below. Please do not edit.]

Reviewers' comments:

Reviewer's Responses to Questions

**Comments to the Author**

1. If the authors have adequately addressed your comments raised in a previous round of review and you feel that this manuscript is now acceptable for publication, you may indicate that here to bypass the “Comments to the Author” section, enter your conflict of interest statement in the “Confidential to Editor” section, and submit your "Accept" recommendation.

Reviewer #1: (No Response)

Reviewer #2: All comments have been addressed

**********

2. Is the manuscript technically sound, and do the data support the conclusions?

The manuscript must describe a technically sound piece of scientific research with data that supports the conclusions. Experiments must have been conducted rigorously, with appropriate controls, replication, and sample sizes. The conclusions must be drawn appropriately based on the data presented. 

Reviewer #1: No

Reviewer #2: Partly

**********

3. Has the statistical analysis been performed appropriately and rigorously? 

Reviewer #1: No

Reviewer #2: N/A

**********

4. Have the authors made all data underlying the findings in their manuscript fully available?

The PLOS Data policy requires authors to make all data underlying the findings described in their manuscript fully available without restriction, with rare exception (please refer to the Data Availability Statement in the manuscript PDF file). The data should be provided as part of the manuscript or its supporting information, or deposited to a public repository. For example, in addition to summary statistics, the data points behind means, medians and variance measures should be available. If there are restrictions on publicly sharing data—e.g. participant privacy or use of data from a third party—those must be specified.

Reviewer #1: No

Reviewer #2: Yes

**********

5. Is the manuscript presented in an intelligible fashion and written in standard English?

PLOS ONE does not copyedit accepted manuscripts, so the language in submitted articles must be clear, correct, and unambiguous. Any typographical or grammatical errors should be corrected at revision, so please note any specific errors here.

Reviewer #1: No

Reviewer #2: Yes

**********

6. Review Comments to the Author

Please use the space provided to explain your answers to the questions above. You may also include additional comments for the author, including concerns about dual publication, research ethics, or publication ethics. (Please upload your review as an attachment if it exceeds 20,000 characters)

Reviewer #1: 1. The background section is still poor. First, it starts at too high a level by trying to convince us why computers are important. Second, papers are discussed briefly and simply placed in a table format, one sentence each, which is not enough to understand how this work is different from other papers which have attempted to compare software models and methodologies.

2. There is almost zero discussion of each results Figure. The manuscript does not tell the readers what the results imply in each plot. Because of this and the lack of y-axis labels in the plots, it is unclear how the authors reached their conclusions regarding the efficacy of different models.

3. Does the distinction between "methodology" and "model" imply that the two are orthogonal? For example, can you have a heavyweight agile combination? or a lightweight waterfall combination? it certainly appears like the two are NOT orthogonal. If that's the case, what's the point of this distinction?

4. It seems unreasonable to me to expect that participants in small organizations can have strong experience with large-scale projects, and vice-versa. How do the authors control for this in their statistical analysis? and how do they control for the respondent's programming experience?

5. Results:

- What is the distribution of software models that the respondents have selected (i.e., how many selected waterfall, AZ, agile, etc)?

- Plots are still missing y-axis labels.

- Error bars appear to be identical in length for same colored bars in each plot. Why is that?

6. To summarize their results, I suggest using a star diagram similar to Figure 2, to show where each model's strengths lie.

7. Language:

The authors should re-examine their manuscript's language. There are clear typos, grammatical errors, and weird phraseology everywhere. A few examples:

- "After completing the paling phase ..." (page 3) -> After completing the planning phase ...

- " the iterative model adopts which is mainly focus on the coding and testing phase" (page 5) -> the iterative model focuses mainly on coding and testing phases

- "Now in the modern year," (page 8)

- "Questioner related to schedule" (Table 4) -> "Questions related to schedule"

8. Software development models are still unnecessarily listed in a bulleted list in page 6.

9. Data availability: the authors include summary data (Table 6) in the supplementary file but it is unclear what the numbers in the table mean. Proper data availability for this manuscript means that the authors must include all survey results (taking care to anonymize the authors and organizations of course).

While the manuscript has improved in several aspects, it still has not addressed the poor writing and fundamental lack of (i) a proper analysis of related literature, (ii) rigorous statistical analysis, and (iii) convincing discussion and conclusions.

Reviewer #2: (No Response)

**********

7. PLOS authors have the option to publish the peer review history of their article (what does this mean?). If published, this will include your full peer review and any attached files.

If you choose “no”, your identity will remain anonymous but your review may still be made public.

**Do you want your identity to be public for this peer review?** For information about this choice, including consent withdrawal, please see our Privacy Policy.

Reviewer #1: No

Reviewer #2: No

[NOTE: If reviewer comments were submitted as an attachment file, they will be attached to this email and accessible via the submission site. Please log into your account, locate the manuscript record, and check for the action link "View Attachments". If this link does not appear, there are no attachment files.]

While revising your submission, please upload your figure files to the Preflight Analysis and Conversion Engine (PACE) digital diagnostic tool, https://pacev2.apexcovantage.com/. PACE helps ensure that figures meet PLOS requirements. To use PACE, you must first register as a user. Registration is free. Then, login and navigate to the UPLOAD tab, where you will find detailed instructions on how to use the tool. If you encounter any issues or have any questions when using PACE, please email PLOS at figures@plos.org. Please note that Supporting Information files do not need this step.

# Author comment: Author response to Decision Letter 1

**DOI:** 10.1371/journal.pone.0264420.r004

24 Aug 2021

Concern 1:: The background section is still poor. First, it starts at too high a level by trying to convince us why computers are important. Second, papers are discussed briefly and simply placed in a table format, one sentence each, which is not enough to understand how this work is different from other papers which have attempted to compare software models and methodologies.

Author response: Back ground description in literature review section is revised according to reviewer’s suggestion.

Author actions: Back ground description in literature review section is revised according to reviewer’s suggestion. Bulleted lists and irrelevant headings are removed. First para about computers introduction is also removed. Remaining paragraphs are also revised/rephrased and proof read. Difference of previous study with this study is also explained on page 6 after Table 2.

Concern 2: There is almost zero discussion of each results Figure. The manuscript does not tell the readers what the results imply in each plot. Because of this and the lack of y-axis labels in the plots, it is unclear how the authors reached their conclusions regarding the efficacy of different models.

Author response: We have updated the results figures discussion section and also plot y-axis labels properly. The resulting figure clearly shows that the comparison of small medium and large-scale projects concerning adopted methodology.

Author actions: We update the results figure section with a detailed discussion also update y-axis labels.

Concern 3: Does the distinction between "methodology" and "model" imply that the two are orthogonal? For example, can you have a heavyweight agile combination? or a lightweight waterfall combination? it certainly appears like the two are NOT orthogonal. If that's the case, what's the point of this distinction?

Author response: A model provides an environment to implement a framework. A methodology is a tool that helps to perform some action. In our research work, our adopted methodology factors judge different software development models based on heavyweight as well as lightweight by factor wise for example budget factor (agile vs waterfall) budget analyze of both models in light as well as heavyweight in a sequential manner.

Author actions: We compare two models with respect to Small Medium and large scale projects also evaluate these models in lightweight as well as heavyweight methodologies.

Concern 4: It seems unreasonable to me to expect that participants in small organizations can have strong experience with large-scale projects, and vice-versa. How do the authors control for this in their statistical analysis? and how do they control for the respondent's programming experience?

Author response: In the data collection method, we explain our respondents have experienced people even in organizations small or large. Respondent programming experience control in questionnaire form.

Author actions: we update the data collection method on page 10.

Concern 5: What is the distribution of software models that the respondents have selected (i.e., how many selected waterfalls, AZ, agile, etc)?

- Plots are still missing y-axis labels.

- Error bars appear to be identical in length for same-colored bars in each plot. Why is that?

Author response: We add the distribution of software models that the respondents have selected in the form of a table in the supporting file also plot the y-axis labels.

Author actions: We update the data collection method section on page 10 and also update the result section figure on page 22.

Concern 6: To summarize their results, I suggest using a star diagram similar to Figure 2, to show where each model's strengths lie.

Author response: We use a star diagram summarize the result results adequately.

Author actions: we update the resulting figure on page 22.

Concern 7: The authors should re-examine their manuscript's language. There are clear typos, grammatical errors, and weird phraseology everywhere. A few examples:

- "After completing the paling phase ..." (page 3) -> After completing the planning phase ...

- " the iterative model adopts which is mainly focus on the coding and testing phase" (page 5) -> the iterative model focuses mainly on coding and testing phases

- "Now in the modern year," (page 8)

- "Questioner related to schedule" (Table 4) -> "Questions related to schedule"

Author response: Manuscript is revised to improve writing and typos.

Author actions: Manuscript is revised to improve writing and typos.

Concern 8: Software development models are still unnecessarily listed in a bulleted list in page 6.

Author response: Bullets are removed as advised. 

Author actions: Bullets are removed as advised.

Concern 9: Data availability: the authors include summary data (Table 6) in the supplementary file but it is unclear what the numbers in the table mean. Proper data availability for this manuscript means that the authors must include all survey results (taking care to anonymize the authors and organizations of course).

Author response: We have update the required table descriptions.

Author actions: we update the description about table 6 and as well as table 7 on page 12.

Once again, authors are grateful to all reviewers for their valuable time and suggestions to improve the manuscript.

Attachment Submitted filename: Responses to reviewers v 1.2.docx Click here for additional data file.

# Peer review: Decision Letter 2

**DOI:** 10.1371/journal.pone.0264420.r005

**Authors:** M. Usman Ashraf

20 Oct 2021

PONE-D-21-05435R2Statistical Analysis of Software Development Models and Six-Pointed Star MethodologyPLOS ONE

Dear Dr. SARWAR,

Thank you for submitting your manuscript to PLOS ONE. After careful consideration, we feel that it has merit but does not fully meet PLOS ONE’s publication criteria as it currently stands. Therefore, we invite you to submit a revised version of the manuscript that addresses the points raised during the review process.

Please submit your revised manuscript by Dec 04 2021 11:59PM. If you will need more time than this to complete your revisions, please reply to this message or contact the journal office at plosone@plos.org. When you're ready to submit your revision, log on to https://www.editorialmanager.com/pone/ and select the 'Submissions Needing Revision' folder to locate your manuscript file.

Please include the following items when submitting your revised manuscript:

- A rebuttal letter that responds to each point raised by the academic editor and reviewer(s). You should upload this letter as a separate file labeled 'Response to Reviewers'.
- A marked-up copy of your manuscript that highlights changes made to the original version. You should upload this as a separate file labeled 'Revised Manuscript with Track Changes'.
- An unmarked version of your revised paper without tracked changes. You should upload this as a separate file labeled 'Manuscript'.

If you would like to make changes to your financial disclosure, please include your updated statement in your cover letter. Guidelines for resubmitting your figure files are available below the reviewer comments at the end of this letter.

If applicable, we recommend that you deposit your laboratory protocols in protocols.io to enhance the reproducibility of your results. Protocols.io assigns your protocol its own identifier (DOI) so that it can be cited independently in the future. For instructions see: https://journals.plos.org/plosone/s/submission-guidelines#loc-laboratory-protocols. Additionally, PLOS ONE offers an option for publishing peer-reviewed Lab Protocol articles, which describe protocols hosted on protocols.io. Read more information on sharing protocols at https://plos.org/protocols?utm_medium=editorial-email&utm_source=authorletters&utm_campaign=protocols.

We look forward to receiving your revised manuscript.

Kind regards,

M. Usman Ashraf, Ph.D

Academic Editor

PLOS ONE

Journal Requirements:

Please review your reference list to ensure that it is complete and correct. If you have cited papers that have been retracted, please include the rationale for doing so in the manuscript text, or remove these references and replace them with relevant current references. Any changes to the reference list should be mentioned in the rebuttal letter that accompanies your revised manuscript. If you need to cite a retracted article, indicate the article’s retracted status in the References list and also include a citation and full reference for the retraction notice.

[Note: HTML markup is below. Please do not edit.]

Reviewers' comments:

Reviewer's Responses to Questions

**Comments to the Author**

1. If the authors have adequately addressed your comments raised in a previous round of review and you feel that this manuscript is now acceptable for publication, you may indicate that here to bypass the “Comments to the Author” section, enter your conflict of interest statement in the “Confidential to Editor” section, and submit your "Accept" recommendation.

Reviewer #1: (No Response)

Reviewer #2: All comments have been addressed

**********

2. Is the manuscript technically sound, and do the data support the conclusions?

The manuscript must describe a technically sound piece of scientific research with data that supports the conclusions. Experiments must have been conducted rigorously, with appropriate controls, replication, and sample sizes. The conclusions must be drawn appropriately based on the data presented. 

Reviewer #1: No

Reviewer #2: Partly

**********

3. Has the statistical analysis been performed appropriately and rigorously? 

Reviewer #1: I Don't Know

Reviewer #2: (No Response)

**********

4. Have the authors made all data underlying the findings in their manuscript fully available?

The PLOS Data policy requires authors to make all data underlying the findings described in their manuscript fully available without restriction, with rare exception (please refer to the Data Availability Statement in the manuscript PDF file). The data should be provided as part of the manuscript or its supporting information, or deposited to a public repository. For example, in addition to summary statistics, the data points behind means, medians and variance measures should be available. If there are restrictions on publicly sharing data—e.g. participant privacy or use of data from a third party—those must be specified.

Reviewer #1: Yes

Reviewer #2: Yes

**********

5. Is the manuscript presented in an intelligible fashion and written in standard English?

PLOS ONE does not copyedit accepted manuscripts, so the language in submitted articles must be clear, correct, and unambiguous. Any typographical or grammatical errors should be corrected at revision, so please note any specific errors here.

Reviewer #1: No

Reviewer #2: Yes

**********

6. Review Comments to the Author

Please use the space provided to explain your answers to the questions above. You may also include additional comments for the author, including concerns about dual publication, research ethics, or publication ethics. (Please upload your review as an attachment if it exceeds 20,000 characters)

Reviewer #1: Dear Editor,

While I appreciate the authors’ efforts in addressing my earlier concerns, I still find that the manuscript suffers from fundamental issues that have not been addressed by the authors and thus, cannot recommend it for acceptance.

First, the authors make an artificial distinction between a “methodology” and a “model”. The way they define it, one can have a “heavyweight agile” combination or a “lightweight waterfall” combination. None of these combinations make any sense; an agile methodology is lightweight by definition. This problematic distinction exists in the survey used to gather the programmers’ feedback. Specifically, the survey asks respondents to indicate the software methodology followed in their company (agile, waterfall, etc), then proceeds to ask them a series of questions, each requiring them to differentiate between a “lightweight” and “heavyweight” methodology. As stated previously, this survey structure means that one can have heavy agile or light waterfall methods, which is nonsensical. The authors’ abbreviated response to this concern was not satisfactory. It would have been better to group the software development models into the two categories instead. For example, the agile model belongs to the “lightweight” category, while the waterfall model belongs to the “heavyweight” category.

Second, the survey assumes that all programmers have experience with both “heavyweight” and “lightweight” methodologies. Again, the authors response to this concern was not satisfactory. They stated that all respondents had 3-5 years’ experience. But that doesn’t mean that all of them have experience with small- and large-scale projects or lightweight and heavyweight methodologies. For example, programmers might be highly experienced but work within small development shops which might favor lighter methods, and vice versa. The survey only asks how many years’ experience the respondent has, not the size of projects they have been involved in the past. This casts serious doubt on the results of the survey as it is highly likely that respondents are only responding with what they perceive to be the advantages and disadvantages of lightweight and heavyweight methodologies, rather than what the respondents experienced when following those methodologies.

Third, it seems to me that some aspects of the 6-star evaluation methodology can be better served by using objective measures rather than surveys. Scheduling, budget, and resources can be measured objectively in terms of time, money, and capital spent. Quality can be measured in terms of unit-test coverage, number of bugs, post-release issues, cyclomatic complexity, etc.

Fourth, the questions relating to scope and risk in the survey are very vague. For example, in the scope section there is this question: “Does the project have a decisive scope by adopting a lightweight methodology”. What does decisive scope mean? How can the respondent know? Similarly, what is meant by meeting business aspirations under the risk section?

While I would have liked to see the revised Figures in revision 2, I couldn’t download them and they weren’t included with the PDF. But those revisions are unlikely to change the fundamental un-addressed problems above.

In short, the methodology and survey adopted by the paper suffers from fundamental problems that make it impossible for me to recommend it for acceptance. To be clear, I don't think my decision will change without properly re-collecting the data using better instruments.

A minor note: the authors made available all the original survey responses on Google drive which is great. But they are not anonymized! the full names of participants and companies are visible.

Reviewer #2: Even though this manuscript has a specific subject of research I think that the presented results can be used as an applied approach to analysis. There are no other special comments to authors.

**********

7. PLOS authors have the option to publish the peer review history of their article (what does this mean?). If published, this will include your full peer review and any attached files.

If you choose “no”, your identity will remain anonymous but your review may still be made public.

**Do you want your identity to be public for this peer review?** For information about this choice, including consent withdrawal, please see our Privacy Policy.

Reviewer #1: No

Reviewer #2: No

[NOTE: If reviewer comments were submitted as an attachment file, they will be attached to this email and accessible via the submission site. Please log into your account, locate the manuscript record, and check for the action link "View Attachments". If this link does not appear, there are no attachment files.]

While revising your submission, please upload your figure files to the Preflight Analysis and Conversion Engine (PACE) digital diagnostic tool, https://pacev2.apexcovantage.com/. PACE helps ensure that figures meet PLOS requirements. To use PACE, you must first register as a user. Registration is free. Then, login and navigate to the UPLOAD tab, where you will find detailed instructions on how to use the tool. If you encounter any issues or have any questions when using PACE, please email PLOS at figures@plos.org. Please note that Supporting Information files do not need this step.

# Author comment: Author response to Decision Letter 2

**DOI:** 10.1371/journal.pone.0264420.r006

13 Dec 2021

Dear reviewers,

The authors owe gratitude to the referees for their valuable comments and suggestions which have helped us to improve the presentation of this paper. Every desirable and necessary changes have been incorporated in the revised version. Please find below the rebuttal of our paper.

Concern 1:

First, the authors make an artificial distinction between a “methodology” and a “model”. The way they define it, one can have a “heavyweight agile” combination or a “lightweight waterfall” combination. None of these combinations make any sense; an agile methodology is lightweight. This problematic distinction exists in the survey used to gather the programmers’ feedback. Specifically, the survey asks respondents to indicate the software methodology followed in their company (agile, waterfall, etc), then proceeds to ask them a series of questions, each requiring them to differentiate between a “lightweight” and “heavyweight” methodology. As stated previously, this survey structure means that one can have heavy agile or light waterfall methods, which is nonsensical. The authors’ abbreviated response to this concern was not satisfactory. It would have been better to group the software development models into the two categories instead. For example, the agile model belongs to the “lightweight” category, while the waterfall model belongs to the “heavyweight” category.

Author response: A model is templete while methodology is study of method that is used in a field.In the updated manuscript the lightweight and heavyweight methodologies are discussed separately. The comparative analysis of lightweight vs lightweight and heavyweight vs heavyweight are discussed separately. The waterfall model belongs to heavyweight methodology so its comparison is done only with the models that belong to heavyweight methodology. The lightweight methodology (agile, AZ) comparison is done within lightweight boundaries. For the categorization of small medium and large scale projects, there is table 7 added to the manuscript. 

Author actions: We have updated the manuscript.

Concern 2:

Second, the survey assumes that all programmers have experience with both “heavyweight” and “lightweight” methodologies. Again, the authors response to this concern was not satisfactory. They stated that all respondents had 3-5 years’ experience. But that doesn’t mean that all of them have experience with small- and large-scale projects or lightweight and heavyweight methodologies. For example, programmers might be highly experienced but work within small development shops which might favour lighter methods, and vice versa. The survey only asks how many years’ experience the respondent has, not the size of projects they have been involved in the past. This casts serious doubt on the results of the survey as it is highly likely that respondents are only responding with what they perceive to be the advantages and disadvantages of lightweight and heavyweight methodologies, rather than what the respondents experienced when following those methodologies.

Author response: For the categorization of small medium and large scale projects, there is table 7 added to the manuscript. In the updated survey questionnaire form the experience section is divided into further subsections (lightweight and heavyweight or both) respondent give their opinion about experiences in lightweight and heavyweight methodologies. The questions relating to scope and risk in the survey are revised. Survey Questioner updated according to reviewer recommendation 

Author actions: We have updated the manuscript.

Concern 3:

Third, it seems to me that some aspects of the 6-star evaluation methodology can be better served by using objective measures rather than surveys. Scheduling, budget, and resources can be measured objectively in terms of time, money, and capital spent. Quality can be measured in terms of unit-test coverage, number of bugs, post-release issues, cyclomatic complexity, etc.

Author response: In 6-star evaluation methodology (Scheduling, budget, and resources) are measured in term of time money and capital but quality measured in term of customer satisfaction and success of project.

Author actions: We have updated the manuscript.

Concern 4:

Fourth, the questions relating to scope and risk in the survey are very vague. For example, in the scope section there is this question: “Does the project have a decisive scope by adopting a lightweight methodology”. What does decisive scope mean? How can the respondent know? Similarly, what is meant by meeting business aspirations under the risk section?

Author response: The questions relating to scope and risk in the survey are revised. Survey Questioner updated according to reviewer recommendation 

Author actions: We have updated the manuscript.

Once again, authors are grateful to all reviewers for their valuable time and suggestions to improve the manuscript.

Attachment Submitted filename: Responses to reviewers v 1.3.docx Click here for additional data file.

# Editor report: Decision Letter 3

**DOI:** 10.1371/journal.pone.0264420.r007

**Authors:** M. Usman Ashraf

11 Feb 2022

Statistical Analysis of Software Development Models by Six-Pointed Star Framework

PONE-D-21-05435R3

Dear Dr. SARWAR,

We’re pleased to inform you that your manuscript has been judged scientifically suitable for publication and will be formally accepted for publication once it meets all outstanding technical requirements.

Within one week, you’ll receive an e-mail detailing the required amendments. When these have been addressed, you’ll receive a formal acceptance letter and your manuscript will be scheduled for publication.

An invoice for payment will follow shortly after the formal acceptance. To ensure an efficient process, please log into Editorial Manager at http://www.editorialmanager.com/pone/, click the 'Update My Information' link at the top of the page, and double check that your user information is up-to-date. If you have any billing related questions, please contact our Author Billing department directly at authorbilling@plos.org.

If your institution or institutions have a press office, please notify them about your upcoming paper to help maximize its impact. If they’ll be preparing press materials, please inform our press team as soon as possible -- no later than 48 hours after receiving the formal acceptance. Your manuscript will remain under strict press embargo until 2 pm Eastern Time on the date of publication. For more information, please contact onepress@plos.org.

Kind regards,

M. Usman Ashraf, Ph.D

Academic Editor

PLOS ONE

Additional Editor Comments (optional):

Authors have addressed all the comments as per the directions. Thank you for considering the constructive comments given by the reviewers. 

# Editor report: Acceptance letter

**DOI:** 10.1371/journal.pone.0264420.r008

**Authors:** M. Usman Ashraf

11 Mar 2022

PONE-D-21-05435R3 

Statistical Analysis of Software Development Models by Six-Pointed Star Framework 

Dear Dr. Sarwar:

I'm pleased to inform you that your manuscript has been deemed suitable for publication in PLOS ONE. Congratulations! Your manuscript is now with our production department. 

If your institution or institutions have a press office, please let them know about your upcoming paper now to help maximize its impact. If they'll be preparing press materials, please inform our press team within the next 48 hours. Your manuscript will remain under strict press embargo until 2 pm Eastern Time on the date of publication. For more information please contact onepress@plos.org.

If we can help with anything else, please email us at plosone@plos.org. 

Thank you for submitting your work to PLOS ONE and supporting open access. 

Kind regards, 

PLOS ONE Editorial Office Staff

on behalf of

Dr. M. Usman Ashraf 

Academic Editor

PLOS ONE
